# Exploring the combined effect of fermentation time, and first drying temperature on black tea flavor profile characterized by GC–MS and GC-IMS

**DOI:** 10.1016/j.fochx.2026.103975

**Published:** 2026-05-12

**Authors:** Muhammad Yasir, Yanhua Jiang, Noman Walayat, Jameel M. Al-Khayri, Mohammed I. Aldaej, Muneera Q. Al-Mssallem, Fatima M. Alessa, Zhucheng Su, Mustafa I. Almaghasla, Ran Wei

**Affiliations:** a Department of Tea Science, Zhejiang Agriculture and Forestry University, Hangzhou 311300, China; b Agricultural Industrialization Development Center of Ninghai County, Ningbo 315699, China; c Department of Agricultural Biotechnology, College of Agriculture and Food Sciences, King Faisal University, Al-Ahsa 31982, Saudi Arabia; d Department of Food Science and Nutrition, College of Agriculture and Food Sciences, King Faisal University, Al-Ahsa 31982, Saudi Arabia; e Plant Pests and Diseases Unit, College of Agriculture and Food Sciences, King Faisal University, Al-Ahsa 31982, Saudi Arabia

**Keywords:** Fermentation, First drying, Sensory evaluation, Black tea processing

## Abstract

Fermentation time and first drying temperature play key roles in black tea flavor quality. We investigated their combined effects on biochemical and volatile compounds (VOCs) in two cultivars. A Fermentation duration of 2.5 h followed by first drying at 95 °C was identified critical point for aroma formation, since additional fermentation and elevated drying temperature declined aroma quality. Prolonged processing decreased free amino acids, reducing sugars, and theaflavin. Gas chromatography mass spectrometry and gas chromatography-ion mobility spectrometry identified 92 and 82 VOCs, primarily alcohols and aldehydes. Key compounds, including cedrol, nonanal, trans-beta-ionone, 2,3-diethyl-5-methylpyrazine, and phenylacetaldehyde contributed to improved aroma of both cultivars subjected to 2.5 h fermentation and 95 °C drying. VOCs based hierarchical clustering revealed that, despite inherent metabolic composition differences in both cultivars the processing conditions produced distinct directional changes. This study provides new insight into dynamic variations driven by combined effect of processing parameters in different cultivars.

## Highlights

• Fermentation and drying synergistically drives tea biochemical and sensory quality
• Prolonged fermentation and high temperature drying decreased black tea quality
• Fermentation for 2.5 h and drying at 95 °C improved black tea quality
• Alcohol and aldehydes were the most abundant VOCs
• Cedrol, nonanal, linalool, trans-β-ionone are crucial for aroma formation

## 1 Introduction

Black tea accounting for up to 75% of global tea consumption has become one of the most consumed non-alcoholic drinks due to its unique flavor and health benefits, including antidiabetic, antioxidants and antimicrobial effects (Su et al., 2024). Several factors such as cultivars type, environmental variables, processing methods and storage conditions mediate the quality of black tea (Zheng et al., 2016). Fermentation catalyzed by polyphenol oxidase (PPO) and peroxidase (POD) is the key step of black tea processing and involves the oxidation of polyphenolic compounds. This process leads to the formation of water soluble pigments including theaflavin (TF), thearubigins (TR) and theabrownins (TBs), which contribute to the color and flavor (Xiao et al., 2020). During processing the tea leaves are macerated to commence fermentation thereby converting catechins into quinones using molecular oxygen (Matsuo & Tanaka, 2017). Due to the difference in reduction potential, quinones contribute to the redox equilibration reactions during fermentation, resulting in various catechins decreasing at different rates (Ngure et al., 2009). Consequently, catechin contents and composition vary as fermentation proceeds thereby changing the color and flavor attributes (Obanda et al., 2001). Following fermentation, first drying is key factor modulating black tea quality. The drying halts fermentation by denaturing oxidation enzymes resulting in locking biochemical changes and reducing moisture content to 3–4%, thereby improving taste and shelf life of black tea (Vargas & Vecchietti, 2016). However, the chemical changes are not instantly stopped; instead, they are initially expedited as the temperature increases, until the unavailability of water to the enzymes or complete enzyme inactivation. Drying at too low temperature can retain the moisture in leaves thereby partial inactivation of oxidation enzymes and continued fermentation, potentially degrading tea quality. Conversely, drying at too high temperature can degrade heat sensitive aroma compounds, polyphenols and amino acids, thereby reducing tea quality. Therefore, it is pertinent to find the suitable fermentation time and drying temperature to produce a uniform-quality black tea. Several studies have reported individual effects of fermentation time and drying temperature on the quality of black tea, however limited attention has been given to understand the combined or synergistic effect of fermentation time and drying temperature on the final quality of black tea. A previous study reported TF formation increased with time during early stages of fermentation of CTC (crush, tear and curl) black tea, reaching maximum at 45 min and changing steadily at 110 min, potentially due to changes in the activity of PPO. The impact of fermentation time and temperature revealed that total TF contents in black tea first increased and then decreased as the fermentation time increased from 60 to 120 min at 20 °C. However, the synthesis and reduction of TF was more pronounced at 30 °C (Obanda et al., 2001). Another finding reported that 3 h fermentation was optimal duration for retaining several catechins, TF, TR as well as synthesis and conversion of sugars and flavonoid degradation in Yunnan congua black tea (Wang, Shen, et al., 2022). Moreover, the abundance of VOCs and umami scores were high in 3 h fermented samples.

Maillard reaction occurs between amino acids and reducing sugars during drying (heating), contributing to the flavor and aroma (Yu et al., 2023). Glucose and L-theanine attributed to the sweetness and umami of black tea are primary monosaccharides and amino acids respectively that take part in Maillard reaction during the drying (Yu et al., 2021), leading to the production of several aroma compounds such as Strecker aldehydes, pyrazine, sulfides, ketones, furans, and several other derivatives. Varying drying temperatures significantly altered the volatile compounds in black tea, such as trans-2-hexenal has found most abundant during drying at 160 °C (Polat et al., 2018). Drying under 100–110 °C has been reported to offer optimal taste and color in black tea, primarily due to formation of Amadori products in the early stage of Maillard reaction, isomerization and degradation processes (Su et al., 2024). Another study employed gas chromatography–mass spectrometry (GC–MS) and gas chromatography ion mobility mass spectrometry (GC-IMS) to detect volatile organic compounds (VOCs) and deciphered ethanol, diacetyl, 2-methl-2-pentanal dimer and 2-methylbutyl acetate as key VOCs causing aroma variation during varying drying conditions. GC–MS is a well-established approach often employed for separation and detection of complex compounds, its specificity, high resolution and extensive database have established its role in various applications including food, beverage, pesticides, and metabolomics analysis (Vu-Duc et al., 2025). In addition, GC-IMS attributed with high throughput detection can characterize ionized molecules at atmospheric temperature and pressure. The use of GC-IMS, a high throughput technique that analyzes ionized molecules at atmospheric temperature and pressure is increasingly used for food flavor profiling, authentication and classification (Wang et al., 2020). However, neither GCMS nor GCIMS alone serve the purpose to comprehensively resolve tea aroma complexity due to inherent tradeoffs between identification rigor and detection sensitivity. GCMS enables high confidence detection and relatively more accurate quantification of key VOCs, whereas GCIMS offers enhanced sensitivity for trace and low abundance compounds, and resolve structurally similar compounds including monomers and dimers. The combination of these techniques allows a more comprehensive and complementary characterization of aroma profiles. Therefore, we employed both approaches to characterize VOCs associated with black tea aroma.

Prior studies have revealed the individual effect of fermentation time and drying temperatures in shaping the flavor of black tea, however the combined effect of fermentation time and drying temperature on the final quality of black tea remains largely unexplored. In the current study we mainly focused on synergistic effect of varying fermentation times (2.5 h, 4 h 5.5 h) followed by first drying temperature treatments (95 °C, 110 °C, and 125 °C) on the final quality of black tea from clonal propagated Wanghai-1 and seed/sexually propagated JiuKeng cultivars. This study employed sensory evaluation, assessment of important biochemical constituents, GC–MS, and GC-IMS for detection of VOCs to explore the underpinnings of black tea flavor profile. This study not only provide robust theoretical basis for optimizing the combined effect of fermentation durations and subsequent drying temperature in black tea processing but also offered potential scientific insights to its industrial scale production.

## 2 Materials and methods

### 2.1 Chemical reagents

Chemicals including foline-phenol, sodium carbonate, potassium dihydrogen phosphate, sodium nitrite, ethanol, and glucose (purity≥99%) were purchased from Sinopharm Chemical Reagent (Shanghai, China). Ninhydrin hydrate (purity≥98%), and anthrone were purchased from Macklin (Shanghai, China). Ethyl decanoate (purity≥99.99%) from Aladdin Biochemical Technology Co., Ltd. (Shanghai, China) was used as internal standard for GC–MS analysis. Methanol, acetonitrile, formic acid, and acetic acid of HPLC grade were purchased from Fisher Chemical (Massachusetts, USA). The remaining chemicals and reagents utilized were of analytical grade.

### 2.2 Sample preparation

Fresh tea leaves at one bud and two leaf stage of the clonally propagated Wanghai-1 and seed propagated JiuKeng cultivars (*Camellia sinensis* L.) were harvested from Tonglinggang Tea Farm, Ninghai County, Ningbo City, Zhejiang province, China as the experimental material.

**Sample processing and treatment application**

(1) Withering: freshly harvested tea leaves were uniformly spread and withered in an automatic withering tank (Yao Jiang Yuan automatic withering tank) for 18 h with alternate cold and hot air withering and relative humidity of 70–75% until the moisture level of 60–62% was achieved.
(2) Rolling: Following withering, leaves were put into the rolling plant (6CR-65, Zhejiang Green Peak Machinery Co., Ltd., Quzhou City, Zhejiang province, China) for 1.5 h (light 17 min, medium 50 min, heavy 20 min, loose 3 min.)
(3) Fermentation: Rolled leaves were subjected to fermentation/oxidation treatment in a specialized fermentation machine (JY-6CFJ-0.7, Jiayou Machinery Co. Ltd., Fujian, China) at 28 °C and 90% humidity for 2.5 h, 4 h and 5.5 h with an even layer of 10-cm thickness.
(4) First drying-: The fermented leaves were subsequently dried in a hot air dryer JY-6CHZ-9B (Fujian Jia You Machinery Co. Ltd., Fujian, China) at 95 °C, 110 °C and 125 °C for 30 min.
(5) Second drying: after cooling for 12 h, second drying was performed at 90 °C for 30 min using hot air dryer mentioned above.
  The overall treatment combinations were in the following order fermentation time and first-drying temperatures 2.5 h (95 °C, 110 °C, 125 °C), 4 h (95 °C, 110 °C, 125 °C), 5.5 h (95 °C, 110 °C, 125 °C) as given in **Table S 1**. Each treatment combination was prepared in triplicate as independent processing batches, in which tea leaves were processed separately under identical conditions at all processing stages, thus all replicates represent biological replications.

### 2.3 Biochemical profiling, sensory evaluation and detection of black tea liquor color

Total tea polyphenol content was measured by foline-phenol method, free amino acid by ninhydrin method, and soluble sugar by anthrone‑sulfuric acid method as determined in our previous study (Wei et al., 2023). Free amino acid were determined following the terms set forth by the China National Standard System (GB/T 8314–2013). Tea pigments theaflavins (TFs), thearubigins (TRs), and theabrownins (TBs) were determined as described in a previous study (Obanda et al., 2001). All the colorimetric analyses were performed employing a spectrophotometer (752 N, INESA, Shanghai, China).

Sensory evaluation was performed by an expert panel of 10 certified members aged between 30 and 50 years, having at least 4 years of experience. All the tea samples were food-grade to confirm safety. Informed consent of the entire panel was obtained before sensory evaluation. No human ethics committee or formal documentation is available, and no ethical permission was required in college to conduct human sensory evaluation. The appropriate protocols for protecting the rights and privacy of participants were employed, such as no coercion to participate, complete disclosure of study requirements and risks, written consent of the panelists, no release of participants data without their consent, ability to withdraw from study at any moment. Black tea samples (3 g) were brewed in 150 mL boiling water for 5 min (GB/T 23776–2018), and subsequently evaluation was performed for aroma and taste attributes. The tea infusions color was determined using a colorimeter (CR5, 3nh Technology, Shanghai, China) with ΔL, Δa, Δb, and ΔE denoting light-dark, red-green, yellow-blue and overall color difference respectively.

### 2.4 Determination of VOCs with GC–MS

The analysis of VOCs from black tea samples was carried out using a gas chromatography–mass spectrometry (GC–MS) analyzer (7890B/7000C, Agilent Technologies, CA, USA). The quantification of VOCs was conducted with subtle changes to the previous study (Yang et al., 2024). Black tea sample (0.5 g) was placed in a 20 mL headspace vial. Afterward, 5 mL boiling water and 10 μL ethyl decanoate (10 mg/L, internal standard) were added, the vial was immediately sealed and allowed to stand for 5 min until the material equilibrium. The manual fibers (DVB/CAR/PDMS, 50/30 μm, Supelco, CA, USA) absorbed volatiles at 60 °C for 60 min. The fibers were then inserted into the GC and thermally desorbed at 250 °C for 5 min. The separation of volatiles was achieved employing an HP-5 ms capillary column (60 m × 250 μm × 0.25 μm, Agilent Technologies, USA), the column temperature was adjusted at 30 °C. The carrier gas was high-purity helium (99.9995%) with a flow rate of 1 mL/min. The temperature program was configured as follows: Started at 40 °C for 5 min, gradually rose to 100 °C at 6 °C/min (with a 2-min hold), and finally to 270 °C at 5 °C/min (with a 4-min hold). The mass spectrometer operated in electron ionization mode with an extended scanning range of 33 *m*/*z* to 550 m/z. The ion source and transmission line temperatures were set at 230 °C and 270 °C, respectively. To conduct both qualitative and quantitative analysis, the retention index (RI) of target compounds was computed using eq. 1. The detected compounds were cross-referenced with the relevant standard compounds listed in the mass spectrometry library (NIST 17 database) and available references. To be more precise, the RI of the target compounds was determined by consulting the n-alkanes (C6–C24) standard curve, which was created using the same GC–MS parameters.

(1) $$\text{Retention index}\;\left(\mathrm{RI}\right)\;\mathrm{RI}=100n+100\left(\frac{t_{i}-t_{n}}{t_{n+1}-t_{n}}\right)$$

Whereas n represents the number of carbon atoms in n-alkanes (C6–C24); *t*_*i*_ represents the retention period of the compound, *t*_*n*_ denotes the retention time of the compound's preceding carbon label, *t*_*n+*1_ refers to the retention time of the next carbon label compound. Relative contents were calculated as described in previous study (Lin et al., 2024).

### 2.5 Determination of VOCs with GC-IMS

Further analysis of VOCs was performed employing GC-IMS Coupler Flavor Spec1H1–00053 G.A.S., Germany). An autosampler (CTC Analytics AG, Zwingen Switzerland) was employed for the extraction of VOCs. Wax capillary column (35 m × 0.53 mm × 1 μm) kept at 60 °C was used for chromatic separation. A 2 g of tea samples and 10 μL of 2-methyl-3-heptanone internal standard (concentration 0.1 μg/mL) were added in a 20 mL headspace vial, after incubation at 60 °C for 20 min at a stirring speed of 500 rpm, 500 μL gas was then injected with an airtight syringe maintained at 85 °C. The IMS was maintained at 45 °C. Nitrogen gas (N_2_) was employed as carrier gas. The programmed flow rate was set as follows; initially, the flow rate was 2 mL/min for 2 min, then ramped to 10 mL/min by 8 min, following ramped to 100 mL/min for the next 10 min, eventually held for 20 min. The carrier gas moved at the constant flow rate of 150 mL/min. The retention index of each compound was calculated, moreover, all VOCs were qualitatively assessed by comparing RI and drift time to the given standards in the IMS and NIST databases. The values of VOCs are relative concentrations. Spectrograms, visualization of two and three-dimensional top views, and fingerprint comparison were performed as described in previous study (Wang et al., 2023).

### 2.6 Statistical analysis

All results are expressed as mean ± standard deviation of three biological replicates. SPSS Statistics (version 26.0, IBM, NY, USA) was used for analysis of variance (ANOVA) and post hoc test (*p* < 0.05). Origin software (2021 Origin Lab, MA, USA) was used for data visualization. SIMCA 14.1 (Umetrics AB, Umea, Sweden) was employed for orthogonal partial least squares discriminant (OPLS-DA) analysis.

## 3 Results and discussion

### 3.1 Variations in color of black tea infusions

The combined effect of fermentation time and first drying temperature significantly influenced infusion color. In WBT samples, Δa (redness) values ranged from 0.9 to 2.0, and peaked at 2.0 ± 0.36 in B2W9 while B2W2 had the lowest (**Table S2**). B2W3 (1.50) showed moderate Δa value whereas B2W9 (2.00) showed highest. Δb values also increased significantly (*p* < 0.05) with fermentation time ranging from 3.13 ± 0.99 to 5.33 ± 0.38 with highest in B2W9. The variations in ΔL values indicates gradual darkening of tea infusions with increasing oxidation. Less ΔL values represent brighter infusion color associated with TF formation, whereas more negative ΔL corresponds to the increased theabrownin synthesis. B2W3 (−2.07), B2W9 (−2.80) and B2W6 (−3.0) showed higher brightness, suggesting improved TF formation and brighter infusion color. In contrast, elevated fermentation time (5.5 h) and subsequent high drying temperature (110 °C) in B2W8 (−4.17) showed darker infusion color, suggesting elevated oxidation and polymerization. This indicated higher accumulation of thearubigin and theabrownin. Similarly, B2W1 and B2W5 (−3.60) showed darker infusion color. This suggest that prolonged fermentation and higher drying temperature decreased tea infusion color quality. B2W3 with least negative ΔL (−2.07) having brightest infusion, moderate Δa and comparatively high Δb, reflects a desirable and balanced bright, red and yellow pigments. This exhibits optimal oxidation and drying, where TF formation was high without excessive conversion into TR and TB. The ΔE indicate overall color change. It is evident (Table S2) that ΔE (5.40) was moderate in B2W3 indicating controlled oxidation and drying avoiding intensive color change. Prolonged oxidation and subsequent high temperature drying in B2W8 and B2W9 showed elevated ΔE values, suggesting pronounced oxidation and pigment transformation. The Δa values also varied significantly (*p* < 0.05) across JBT samples. Overall, Δa values depicted a fluctuating trend, however increased with fermentation time and finally peaked at 5.5 h in combination with drying at 125 °C in B2J7. Whereas, Δb values ranged from 3.53 to 5.27 with an average of 4.39, B2J2 and B2J5 having the lowest and highest values respectively. ΔL values were rather stable with least negative value (−3.73) in B2J6. Similarly, Δa (1.73) and Δb (4.70) were more balanced in B2J6. This suggest that B2J6 exhibited more balanced oxidation and subsequent drying conditions, avoiding excessive darkening and maintaining red and yellow pigments. The processing conditions significantly altered ΔE values (*p* < 0.05). Relatively higher ΔE values in B2W7, B2W8 and B2W9 showed extensive oxidation and polymerization, while lower values depicted mild color development. Notably B2W3 showed intermediate ΔE values. The B2J9 showed higher ΔE values suggesting elevated pigments formation, whereas B2J6 exhibited moderate ΔE and balanced ΔL, Δa and Δb representing better infusion color profile.

### 3.2 The effect of fermentation time and drying temperatures on biochemical attributes

Taste characteristics of black tea have been attributed to the complementary sensation of bitter, umami and sweet, which were induced by associated chemical constituents such as polyphenols, free amino acids and reducing sugars (Su et al., 2024).Tea polyphenols, undergo endogenous enzymatic oxidation, and subsequent non-enzymatic polymerization during fermentation and drying process, forming catechin derived oligomers and polymers, including theaflavins (TFs), thearubigins (TRs) and theabrownins (TBs) which are key quality parameters in the black tea industry (Obanda et al., 2001). Polyphenols, which play key role in developing bitterness and astringency substantially decreased (*p* < 0.05) as the fermentation time and drying temperature increased (Table 1**)**. In WBT samples, polyphenol contents ranged from 6% to 7.80% with B2W3 (7.80 ± 0.09%) exhibiting significantly higher level than B2W2 (7.42 ± 0.15%) and B2W1 (7.16 ± 0.18%). Similarly, B2W9 (6.77 ± 0.23%) contained significantly higher polyphenols compared to B2W8 and B2W7. Significant decline in polyphenol contents between WBT samples fermented for 2.5 h, 4 h, and subsequent drying at 95 °C underscores interactive effect of different fermentation time and drying temperature on polyphenol degradation. Notably, no significant differences were observed among WBT samples fermented for 4 h in combination with subsequent drying at all temperatures. In JBT, the polyphenols ranged from 9.66 ± 0.09% to 10.95 ± 0.15. B2J2 (10.95 ± 0.09%), B2J3 (10.85 ± 0.09), B2J6 (10.42 ± 0.23) and B2J9 (10.25 ± 0.23) were significantly higher (*p* < 0.05). The interactive effect of fermentation times and drying temperatures revealed that polyphenol contents significantly decreased as the fermentation time and drying temperatures increased. The black tea samples of both cultivars fermented at 2.5 h, 4 h and 5.5 h in combination with drying at 95 °C, (B2W3, B2W6,B2W9, B2J3, B2J6 and B2J9) retained significantly higher polyphenol contents. The gradual decline in polyphenol contents with increasing drying temperature indicates thermal sensitivity of these compounds. Our results align with the earlier studies indicating that high drying temperatures and prolonged fermentation significantly reduce polyphenol contents in black tea (Su et al., 2025). The decline in polyphenols with prolonged fermentation followed by drying at elevated temperatures could be attributed to sustained enzymatic oxidation mediated by polyphenol oxidase (PPO) and peroxidase (POD), followed by non-enzymatic polymerization at high temperatures. During early fermentation, catechins are oxidized to theaflavins, which further polymerize into thearubigin and finally theabrownins under prolonged fermentation or elevated temperature. Excessive fermentation or high drying temperatures accelerates this (catechin) conversion process, leading to progressive depletion of measurable polyphenols.

**Table 1:** Combined effect of fermentation time and subsequent drying temperatures on the biochemical composition of black tea samples.

Sample names	Fermentation times	Drying temperatures	Tea polyphenols	Free amino acids	Reducing sugars
B2W1	2.5 h	125 °C	7.16 ± 0.18^b^	5.60 ± 0.13^b^	4.73 ± 0.21^d^
B2W2		110 °C	7.42 ± 0.15^b^	5.79 ± 0.04^b^	4.76 ± 0.07^d^
B2W3		95 °C	7.80 ± 0.09^a^	6.14 ± 0.23^a^	5.58 ± 0.13^a^
B2W4	4 h	125 °C	6.66 ± 0.23^c^	4.82 ± 0.11^d^	5.12 ± 0.03^c^
B2W5		110 °C	6.59 ± 0.09^c^	5.33 ± 0.11^c^	5.08 ± 0.06^c^
B2W6		95 °C	6.71 ± 0.18^c^	5.84 ± 0.06^b^	5.30 ± 0.09^bc^
B2W7	5.5 h	125 °C	6.00 ± 0.00^e^	5.03 ± 0.19^d^	4.49 ± 0.27^e^
B2W8		110 °C	6.29 ± 0.09^d^	5.65 ± 0.13^b^	5.22 ± 0.03^bc^
B2W9		95 °C	6.77 ± 0.23^c^	6.21 ± 0.02^a^	5.43 ± 0.13^ab^
B2J1	2.5 h	125 °C	10.09 ± 0.18^bcd^	4.00 ± 0.16^abc^	6.28 ± 0.09^a^
B2J2		110 °C	10.95 ± 0.15^a^	4.04 ± 0.12^ab^	6.26 ± 0.02^a^
B2J3		95 °C	10.85 ± 0.09^a^	3.98 ± 0.15^abcd^	6.00 ± 0.22^b^
B2J4	4 h	125 °C	10.00 ± 0.18^cd^	3.72 ± 0.10^de^	5.62 ± 0.03^c^
B2J5		110 °C	9.80 ± 0.23^de^	3.75 ± 0.25^cde^	6.02 ± 0.09^b^
B2J6		95 °C	10.42 ± 0.23^b^	4.17 ± 0.12^a^	5.75 ± 0.07^c^
B2J7	5.5 h	125 °C	10.09 ± 0.18^bcd^	3.64 ± 0.07^de^	5.32 ± 0.21^d^
B2J8		110 °C	9.66 ± 0.09^e^	3.80 ± 0.05^bcde^	5.26 ± 0.02^d^
B2J9		95 °C	10.25 ± 0.23^bc^	3.89 ± 0.17^bcde^	5.61 ± 0.06^c^

Different letters in the same column represent significance difference among samples of each cultivar (*p* < 0.05).

After fermentation, the enzymatic activities are terminated by thermal interaction during drying stage of black tea, thereby commencing Maillard and caramelization reactions, which primarily involve amino acids and reducing sugars as substrates (Su et al., 2024). Free amino acids and reducing sugars are dominant taste contributors to the black tea. Moreover, amino acids are key precursors of volatile metabolites such as aldehydes, contributing to the flavor formation of black tea (Hua et al., 2024). The synergistic effect of different combinations of fermentation time and drying temperatures depicted obvious variations in the free amino acid contents, with significantly higher values across all fermentation durations in combination with subsequent drying at 95 °C. In WBT, amino acid contents in B2W3 (6.14 ± 0.23%), B2W6 (5.84 ± 0.06%), and B2W9 (6.21 ± 0.02%) were significantly higher than samples dried at 125 °C (B2W4 and B2W7) across all fermentation durations. This highlights that lower drying temperature significantly retained amino acid contents, whereas higher temperatures exacerbate their degradation, likely via Millard and Strecker reactions. Across all the fermentation durations no linear trend was observed, nonetheless prolonged fermentation combined with higher first drying temperature (B2W4, B2W7) resulted significantly reduced amino acid content, confirming synergistic degradation effects. These findings align with the previous results that prolonged fermentation and drying reduce amino acid contents (Hua et al., 2024). In JBT samples, the overall amino acid was lower with less pronounced variations compared to WBT. B2J6 (4.17 ± 0.12%) had the highest amino acid contents, while B2J4 (3.72 ± 0.10%) and B2J7 (3.64 ± 0.07%) had the lowest values. Unlike WBT, highest free amino acid did not occur at shortest fermentation duration (2.5 h) but at moderate fermentation time (4 h), however the drying temperature combination was again 95 °C. This discrepancy could be attributed to the cultivar specific metabolic response. In addition, this indicates that fermentation time and first drying temperature synergistically influence the amino acid degradation, with no single dominant variable.

Elevated contents of reducing sugars contribute to the mellow taste and floral aroma of black tea. Moreover, the dark color of black tea infusion is closely associated with caramelization of sugars at high temperatures. In WBT, reducing sugars followed similar pattern of amino acid but it was relatively stable. B2W3 (5.58 ± 0.13%) and B2W9 (5.43 ± 0.13%) had the highest levels, whereas B2W6 (5.30 ± 0.09%) was moderately high, all corresponding to 95 °C first drying temperature across all fermentation durations. In contrast B2W1 (4.73 ± 0.21%) and B2W7 (4.49 ± 0.27%), showed significant depletion. These variations clearly exhibits that higher temperatures aggravate sugar depletion through Maillard reaction, while lower drying temperatures favored the sugar retention. In contrast, JBT showed comparatively higher reducing sugar contents than WBT, but followed a clearer declining trend with prolonged fermentation time and higher drying temperature. Interestingly the samples processed across all fermentation times and subsequent drying at 95 °C (B2J3, B2J6 and B2J9) maintained intermediate preservation of sugar contents. The highest values were observed in B2J1 (6.28 ± 0.09b%) and B2J2 (6.26 ± 0.02%), whereas B2J7 (5.32 ± 0.21%) and B2J8 (5.26 ± 0.02%) were significantly lower, highlighting the effect of prolonged fermentation and elevated drying temperature. These results indicate that processing conditions drive consistent directional changes in both cultivars, but the sensitivity and degree differed. WBT showed higher retention of amino acids and sugars at low drying temperature (95 °C), but JBT exhibited overall higher sugar contents but pronounced susceptibility to combined effect of prolonged fermentation and high drying temperatures. The combined effect of varying fermentation durations and first drying at 95 °C was found to better retain the polyphenols, free amino acids and reducing sugars. The reduction in free amino acids and reducing sugars particularly under high drying temperatures is primarily associated with their participation in Millard and Strecker degradation pathways. These reactions are intensified at elevated temperatures, leading to the formation of volatile aroma compounds such as aldehydes, ketones, and pyrazines, but simultaneously depleting precursors responsible for umami and sweetness. Therefore, drying conditions (95 °C) likely preserve these precursors while still enabling controlled aroma formation.

Tea polyphenols particularly tea catechins oxidize during fermentation thereby forming theaflavin (TF), thearubigin (TR), and theabrownins (TB), imparting color and brightness to the tea infusions (Obanda et al., 2001). Theaflavin can improve not only the freshness of flavor but also the brightness of tea infusion. Theaflavin contents were significantly affected by synergistic effect of different combinations of fermentation times and drying temperatures particularly in WBT **(**Table 2**)**. At 2.5 h, fermentation, TF contents were highest at 95 °C drying temperature B2W3 (1.04 ± 0.01%) and decreased with increasing drying temperature. At 4 h, the highest TF contents were found at 95 °C and 110 °C of drying B2W3 and B2W5 (1.07 ± 0.01). However, at prolonged fermentation (5.5 h) TF contents significantly declined with the lowest values at 125 °C in B2W7 (0.83 ± 0.03%). In contrast JBT exhibited minimal variations in TF contents (0.20 ± 0.02% to 0.25 ± 0.02%). This inconsistency might be resulted due to difference of cultivars. During initial stages of fermentation and lower first drying temperature, higher activities of PPO and peroxidase POD promotes the oxidation of catechins into TF, thereby increasing TF contents. However, prolonged fermentation and elevated first drying temperature reduce enzymes stability and further convert TF into TR and TB, leading to decline in TF contents. However, this decline could be attributed to thermal degradation of TF and non-enzymatic conversion into TB and TR. Our findings are in agreement with previous reports showing a significant decrease in TF at higher drying temperatures (Su et al., 2024). Similarly, maximum TF formation occurred after 80 min of fermentation, in black tea, followed by decline at prolonged fermentation, potentially due to reduced activity of PPO (Samanta et al., 2015).

**Table 2:** Combined effect of fermentation time and subsequent drying temperatures on black tea pigments contents (%).

Sample names	Fermentation times	Drying temperatures	Theaflavin	Thearubigin	Theabrownin
B2W1	2.5 h	125 °C	0.92 ± 0.02^cd^	2.42 ± 0.22^ab^	4.63 ± 0.02^d^
B2W2		110 °C	0.97 ± 0.02^bc^	2.51 ± 0.12^ab^	4.66 ± 0.03^d^
B2W3		95 °C	1.04 ± 0.01^a^	2.66 ± 0.43^a^	5.18 ± 0.16^b^
B2W4	4 h	125 °C	0.88 ± 0.04^de^	1.92 ± 0.21^c^	4.49 ± 0.12^e^
B2W5		110 °C	1.07 ± 0.01^a^	2.16 ± 0.03^bc^	5.43 ± 0.03^a^
B2W6		95 °C	1.02 ± 0.01^ab^	1.79 ± 0.08^cd^	5.23 ± 0.04^b^
B2W7	5.5 h	125 °C	0.83 ± 0.03^e^	1.35 ± 0.21^e^	4.16 ± 0.04^e^
B2W8		110 °C	0.90 ± 0.01^d^	1.85 ± 0.10^cd^	4.18 ± 0.01^e^
B2W9		95 °C	0.93 ± 0.01^cd^	1.54 ± 0.11^ce^	4.82 ± 0.04^c^
B2J1	2.5 h	125 °C	0.21 ± 0.04^a^	2.35 ± 0.04^c^	7.98 ± 0.03^b^
B2J2		110 °C	0.21 ± 0.04^a^	3.35 ± 0.20^a^	7.27 ± 0.23^c^
B2J3		95 °C	0.22 ± 0.03^a^	3.14 ± 0.13^a^	7.30 ± 0.10^c^
B2J4	4 h	125 °C	0.20 ± 0.02^a^	2.26 ± 0.10^c^	7.63 ± 0.07^c^
B2J5		110 °C	0.22 ± 0.01^a^	2.73 ± 0.24^b^	7.34 ± 0.08^c^
B2J6		95 °C	0.22 ± 0.01^a^	2.41 ± 0.20^c^	7.47 ± 0.22^c^
B2J7	5.5 h	125 °C	0.25 ± 0.03^a^	2.27 ± 0.07^c^	8.57 ± 0.13^a^
B2J8		110 °C	0.25 ± 0.02^a^	2.72 ± 0.04^b^	7.38 ± 0.09^c^
B2J9		95 °C	0.25 ± 0.03^a^	2.20 ± 0.19^c^	8.03 ± 0.20^b^

Different letters in the same column represent significance of values (p < 0.05) for each cultivar.

During fermentation TF undergo further oxidation thereby forming polymerized TR, contributing to the color, taste and biological activities of black tea (Hua et al., 2024; Huang et al., 2019). In WBT, TR at 2.5 h fermentation duration followed by subsequent drying at 95 °C B2W3 (2.66 ± 0.43%) was highest. The TR contents significantly decreased as the fermentation time increased reaching the significant lowest level in B2W7 (1.35 ± 0.21%). In addition, within each fermentation duration and drying temperature grouping the TR contents generally decreased at the elevated drying temperature. These findings illustrates that prolonged fermentation accelerates further oxidation or polymerization of TR into theabrownins or other derivative compounds, while elevated drying temperatures promote this degradation. TR contents in JBT were consistently higher than in WBT. The highest values were observed in samples processed at shorter fermentation duration (2.5 h) in B2J2 (3.35 ± 0.20%) and B2J3 (3.14 ± 0.13%), whereas samples subjected to prolonged fermentation duration (5.5 h) retained lower TR contents in B2J9 (2.20 ± 0.19). Unlike WBT, medium drying temperature (110 °C) retained higher TR contents, like B2J2 and B2J5, suggesting cultivar specific thermal stability effect. However, the overall decrease across fermentation time remains evident. These findings revealed that TR are intermediate products of oxidation process of black tea, which increase at early to mid-fermentation stage followed by decline at prolonged fermentation time and elevated drying temperature.

Theabrownin contents exhibited significant variations with fermentation time and drying temperature. In WBT, TB content decreased with increasing drying temperature at 2.5 h fermentation, the highest contents reaching the highest level in B2W3 (5.18 ± 0.16%), (Table 2**)**. Whereas, at 4 h, highest TB was found in B2W5 (5.43 ± 0.03%) and B2W6 (5.23 ± 0.04). At 5.5 h, TB contents overall decreased with increase in subsequent drying temperature but remained significantly higher at 95 °C, B2W9 (4.82 ± 0.04%) compared to B2W7 and B2W9. Prior studies have reported that TR significantly reduced as the drying temperature increased from 90 to 110 °C in black tea, though increased beyond 120–140 °C (Peng et al., 2013). In JBT, TR were consistently higher than WBT across all combinations of fermentation duration and first drying temperature. The highest TR values were found in B2J7 (8.57 ± 0.13%) and B2J9 (8.03 ± 0.20%) illustrating accelerated polymerization under prolonged fermentation and was further modulated by elevated drying temperature. Overall, the combined effect of prolonged fermentation time followed by drying at 125 °C resulted in highest TR contents, depicting continuous oxidation and condensation of polyphenol contents. Taken together, the results of polyphenols, amino acids and reducing sugars revealed that optimum contents were retained in samples subjected to fermentation across all time points in combination with 95 °C drying. For further analysis of VOCs, we selected representative samples B2W3, B2W6, B2W9 from WBT, and B2J3, B2J6 and B2J9 from JBT.

### 3.3 Volatile compounds detected by GC–MS

#### 3.3.1 Variation in VOCs due to varying fermentation time followed by drying at 95 °C

**Fig. 1:**
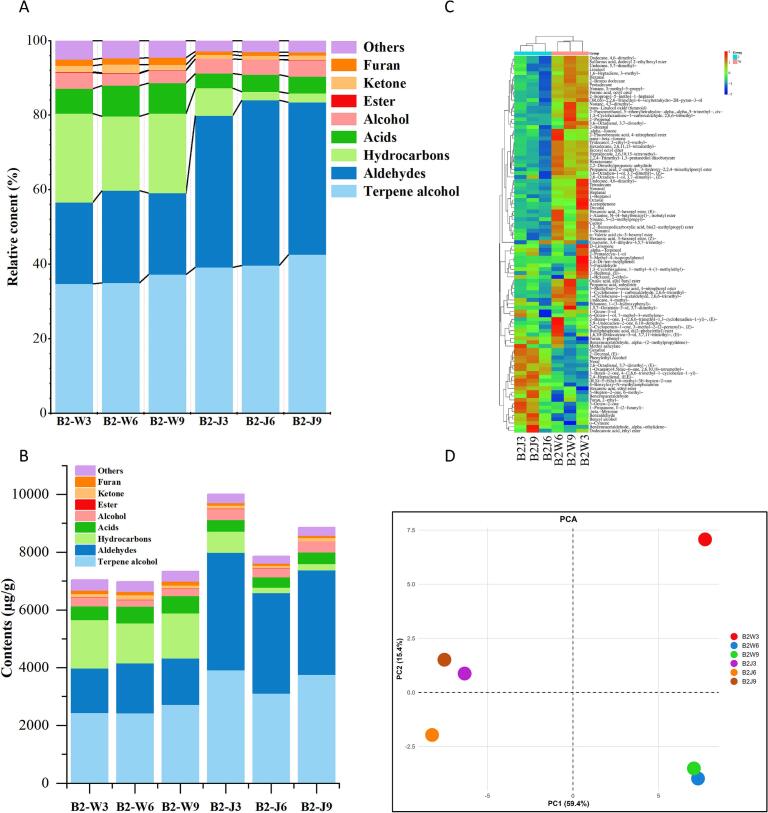
Comparison of VOCs (detected by GC–MS) profiles of black tea samples subjected to varying fermentation time and subsequent drying at 95 °C (A) Composition of relative contents percentage of VOCs species (B) Absolute contents of VOCs (μg/g). (C) Heat map and the hierarchical cluster of the VOCs showing variation trends of VOCs among black tea sample (D) PCA biplot illustrating the separation of tea samples of two cultivars subjected to varying fermentation time followed by drying at 95 °C; clear grouping along PC1 and PC2 indicates distinct VOCs profiles of black tea samples.

A compendium of 98 VOCs was detected across six samples fermented at varying durations followed by drying at 95 °C (Table S3), including 21 aldehydes, 15 acids, 14 hydrocarbons, 11 ketones, 9 alcohols, 9 terpene alcohols, 6 heterocyclic compounds, 3 furans, 2 esters, and 8 others. To show the variations and characteristics of VOCs among different samples of both cultivars, we summarized the relative percent, overall contents and variation trend of each sample, and further performed principal component analysis (PCA) as depicted in Fig. 1. It was observed that percentage of VOCs varied within cultivar and between cultivars (Fig. 1 **A)**. Terpene alcohol was most prevalent (35–43%) followed by aldehyde (22–41%), hydrocarbons (2–21%), acids (4–8%), alcohols (3–4%), and others up to (3–5%), of the total VOCs. Terpene alcohol was most abundant in B2W9 (37%) and B2J9 (43%), whereas aldehydes were highest in B2W6 (25%) and B2J6 (44%). Fig. 1**B** shows that terpene alcohols and aldehydes were consistently dominant in both cultivars. Terpene alcohols reaching highest (2733.73 μg/g) in B2W9 and (3921.08 μg/g) in B2J3 and aldehydes (1726.20 μg/g) in B2W6 and (4074.40 μg/g) in B2J3. The total VOCs varied significantly among treatments and between cultivars. In WBT, the total VOCs was initially high in B2W3, decreased slightly in B2W6 and moderately increased again in B2W9 indicating dynamic variation in VOCs accumulation across the treatments. In contrast, JBT exhibited a pronounced abundance of VOCs reaching highest in B2J3, followed by decline in B2W6 and moderate recovery in B2J9. This trend can be attributed to the balancing of VOCs biosynthesis and degradation pathways. Terpene alcohol was most abundant class of VOCs contributing substantially to the total VOCs profile, particularly in B2W9 and B2J3. During early fermentation (2.5 h). The overall abundance of total VOCs was highest in JBT, suggesting stronger aroma and taste forming potential particularly in B2J3. The non-linear trend of VOC accumulation (increase at 2.5 h, decline at 4 h, and partial recovery at 5.5 h) reflects the dynamic balance between biosynthesis and degradation processes. During early fermentation, enzymatic hydrolysis of glycosidically bound precursors and lipid oxidation promotes rapid formation of alcohols, aldehydes, and terpene derived volatiles. However, prolonged fermentation leads to substrate depletion and secondary transformations, including oxidation, isomerization, and volatilization losses, resulting in reduced VOC abundance. At later stages, partial recovery of certain VOCs may occur due to secondary degradation pathways and thermal release during drying. Similar patterns of VOCs variations have been reported during black tea fermentation, elucidating enzymatic mediated regulation of aroma profile.

Several VOCs exhibited elevated expression across varying fermentation times followed by first drying at 95 °C (Fig. 1**C)**. B2J3 exhibited elevated expression of over 13 VOCs like beta-myrcene (woody, resinous), 1 propanone, 1-(2-furanyl)-, 3-octen-2-one, furan,2-ethyl (smoky) and methyl salicylate (minty, green). Similarly, B2J9 depicted elevated expression of 12 fruity and floral VOCs including benzeneacetaldehyde alpha-ethylidene-, dodecanoic acid ethyl ester, benzaldehyde, benzeneacetaldehyde, neral, and benzeneacetaldehyde alpha-(2-methylpropylidene. Benzeneacetaldehyde, an amino acid derived VOC have shown an increasing trend during black tea fermentation thereby significantly contributing to the aroma profile (Pu et al., 2025) The B2J6 showed substantial expression of 17 compounds including 2-Buten-1-one,1-(2,6,6-trimethyl-1.0.3-cyclohexadien-1-yl)-,(E), 5,9-undecadien-2-one,6,10-dimethyl, 2-cyclopenten-1-one,3-methy1–2-(2-pentenyl)-,(Z), trans-beta-ionone and others imparting floral, fruity and minty notes. The enzymatic oxidation of *β*-carotene by carotenoid cleavage dioxygenase 4 enzyme produce trans-*β-*ionone which is major contributor to the floral aroma of black tea. In WBT, B2W3 showed highest number of significantly accumulated VOCs (21) including, 3-methyl-4-isopropylphenol, 2,4-di-tert-butylphenol (herbal odor), 3-furaldehyde (sweet, caramel aroma), d-limonene (citrus, orange-like), heptanal (green, oily aroma),1-heptanol (green) and octanal having citrus aroma. Notably, these VOCs were exclusively high in B2W3 (2.5 h, 95 °C) as compared to all other samples of both cultivars, indicating potential high aroma. Furthermore, B2W6 and B2W9 exhibited higher expression of 17 and 15 VOCs, respectively. 1,5,7-octatrien-3-ol,3,7-dimethyl-, oxalic acid, allyl butyl ester, 2-butenal, 2-propanal, trans-linalool oxide (floral type odor), nonane 4,5-dimethyl- demonstrated significantly higher accumulation in B2W9. These variations suggest that fermentation followed by first drying at 95 °C significantly altered the VOCs profile of both cultivars, consequently changing the flavor attributes of black tea infusions. Particularly the expression of certain VOCs in specific samples explains that synergistic effect of varying fermentation duration and subsequent first drying at 95 °C significantly affect the quality of tea.

VOCs showed dynamic variations among samples subjected to varying fermentation time and subsequent drying at 95 °C (Fig. 1**C)**. Particularly, expression of more than 27 VOCs in B2J6, 13 in B2J9, and 9 in B2J3 declined. The abundance of more than 10 VOCs in B2W6, 6 in B2W9, and 7 in B2W3 decreased, including o-cymene, benzaldehyde, benzyl alcohol, beta-myrcene, 1 propanone, and benzeneacetaldehyde. These findings suggest that prolonged fermentation combined with subsequent drying at 95 °C led to significant decline in several VOCs, thereby reducing cumulative effect of several aroma active compounds in black tea. The abundance and retention of VOCs in B2W3 exceeded those of B2W6 and B2W9 (Fig. 1**C)**. Similarly, the abundance and retention of most of the volatile compounds was high in B2J3 than B2J6 and B2J9. The superior retention of biochemical components and VOCs at shorter fermentation duration and subsequent drying at 95 °C can be attributed to a balanced enzymatic activation and minimal thermal degradation. Short fermentation duration inhibit excessive activity of PPO and POD thereby preserving catechins, free amino acids, reducing sugars and other biochemical compounds. Whereas, subsequent drying at 95 °C ensures enzyme inactivation to prevent further oxidative degradation, and preservation of thermolabile VOCs and synthesis of desirable aroma compounds through Maillard reaction. Conversely, prolonged fermentation and high drying temperatures likely accelerate excessive oxidation and thermal degradation of key volatile and non-volatile compounds.

Principal component analysis (PCA) was conducted to characterize variation in VOCs profile of both cultivars subjected to three processing conditions (Fig. 1**D)**. The first two principal components explained 74.8% of the total variance, with PC1 (59.4%) and PC2 (15.4%) clearly separating the two cultivars. Both cultivars exhibited significant variation in VOCs in response to treatments, with B2W3 and B2J3 showing clear separation from other samples. This suggests key difference in the volatile compounds retention/degradation in B2W3 and B2J3 compared to other samples.

#### 3.3.2 Screening of key VOCs detected by GC–MS

The overall aroma of black tea is determined by both concentration of VOCs and their aroma threshold (Kraujalytė et al., 2016). To determine key VOCs, we calculated the variable importance in projection (VIP) and odor activity values (OAV). The aroma threshold values were compiled from different resources including the book entitled “*Compilation of Odour Threshold in Air, Water and other Media*” by L.J. Van Gemert (2011) (Van Gemert, 2011). A total of 50 VOCs had VIP > 1 comprising 13 hydrocarbons, 11 aldehydes, and 7 alcohols (**Table S4),** whereas 21 had OAV >1 (**Table S5)**. Twelve key VOCs met both criteria (VIP >1, OAV >1) (Table 3**)**. In WBT, some VOCs significantly declined as fermentation time increased followed by drying at 95 °C. The OAV of cedrol and nonanal 3.47–30.73 consistently decreased with prolonged fermentation, with highest level in B2W3. A similar pattern was observed in B2J3 where OAVs of these VOCs ranged from 1.31 to 12.28, with highest values in B2J3. These findings suggested that despite difference of the cultivar types the combined effect of fermentation time of 2.5 h followed by drying at 95 °C increased the abundance of cedrol (sandalwood notes) and nonanal (floral green lemon-like aroma) contributing to enhanced aroma of B2W3 and B2J3. Nonanal is produced through oxidative degradation of unsaturated fatty acids from lipids during black tea fermentation and thermal processing. Prior studies reported that nonanal increased when tea liquor was heated at 85 °C and 95 °C but decreased as the temperature increased (Kim et al., 2007). Similarly, a previous study reported that VOCs like nonanal with excellent aroma was high during second drying (box hot air 90 °C) of round green tea leaves (Wang, Yu, et al., 2022). An enantiomer is an optical non-superposable mirror images of a molecular entity. Terpene alcohol (3*R*,6*S*)-2,2,6-Trimethyl-6-vinyltetrahydro-2H-pyran-3-ol known as trans-linalool oxide (pyran) is a linalool oxide enantiomer found in black tea imparting leafy aroma. This enantiomer is the product of enzymatic oxidation of linalool (Wang et al., 1994). The OAVs of trans-linalool oxide (pyran) notably varied in both cultivars ranging from 12.9 in B2J6 to 25.9 in B2W3.

**Table 3:** VOCs detected by GC–MS with OAV and VIP > 1.

#	CAS	Compounds	RI	Category	Threshold μg/g	Odor quality	OAV						
							B2W3	B2W6	B2W9	B2J3	B2J6	B2J9	VIP
1	77–53-2	Cedrol	1600	Terpene alcohol	0.5	Cedar, sandalwood note	4.97	4.50	3.47	1.88	1.31	1.85	1.38
2	124–19-6	Nonanal	1104	Aldehyde	2.8	Floral, fatty, green, lemon-like	30.73	22.34	22.22	12.28	9.21	11.38	1.37
3	78–70-6	Linalool	1099	Terpene alcohol	2.8	Floral, sweet, grape-like, woody	12,333.70	11,785.3	13,510.2	8065.0	5838.7	5429.8	1.31
4	66–25-1	Hexanal	801	Aldehyde	4.5	Grassy, green, fresh, fatty	15.20	14.90	17.0	8.70	6.10	9.60	1.31
5	55,722–59-3	3,6-Octadienal, 3,7- dimethyl-	1184	Aldehyde	0.085	Floral-citrusy, delicate odor	837.50	301.10	849.20	50.10	33.50	36.70	1.28
6	39,028–58-5	(3*R*,6*S*)-2,2,6-Trimethyl- 6-vinyltetrahydro- 2H-pyran-3-ol	1173	Terpene alcohol	0.56	Earthy or woody aromas	25.90	22.10	23.50	16.20	12.90	18.30	1.26
7	111–71-7	Heptanal	901	Aldehyde	6.1	Green, oily, grassy	4.40	2.90	3.40	2.00	1.90	2.10	1.24
8	127–41-3	α-Ionone	1426	Ketone	0.4	Floral, violet-like, powdery, berry-like	13.40	19.40	13.50	8.20	6.50	9.80	1.17
9	124–13-0	Octanal		Aldehyde	3.4	Floral and fruity aroma	2.20	1.30	1.40	0.80	0.70	1.10	1.18
10	432–25-7	1-Cyclohexene-1- carboxaldehyde, 2,6,6- trimethyl-	1220	Aldehyde	5	Tropical, saffron, herbal, clean, rose, oxide, sweet, tobacco, damascone, fruity	2.20	3.20	3.50	1.80	1.60	1.80	1.16
11	5989-33-3	2-Furanmethanol, 5-ethenyltetrahydro- .alpha.,.alpha.,5-trimethyl-, cis-	1074	Furan	65	Earthy floral sweet woody	1.80	1.70	2.10	1.30	1.10	1.10	1.13
12	79–77-6	Trans β Ionone	1486	Ketone	0.09	Cedar wood, floral aroma	657.10	1073.30	640.10	406.70	304.5	378.40	1.12

Note: RI represented retention index.

Among several compounds, linalool, a monoterpene alcohol renowned for its characteristic floral aroma is derived from the hydrolysis of *β*-glucosides and *β*-primverosides in tea leaves. It is a key marker of aroma quality in black tea with reported concentrations of (70–241 μg/kg) in Turkish, (77–626 μg/kg) in Indian, and (12.7–2764 μg/kg) in Chinese black tea (Baldermann et al., 2014; Wang, Chen, et al., 2022). Notably, the OAV of linalool was highest among all VOCs detected in both cultivars. In WBT, linalool OAV was high in B2W3 (12,333.7) followed by decrease in B2W6 (11,785.3) reaching its peak in B2W9 (13,510.2). In addition, its OAVs in B2J3 was (8065) and B2J9 (5429). Suggesting that combined effect of prolonged fermentation and drying declined the abundance of linalool in JBT. Oxidative rearrangements convert linalool into linalool oxide and linalool oxide III, moreover during fermentation double bond hydration and cyclization transform linalool into 4-terpineol (Hu et al., 2021). Consequently, prolonged fermentation reduces linalool contents.

Aldehydes produced by oxidative degradation of amino acids (primarily phenylalanine) significantly contribute to the aroma of black tea (Kumazawa & Masuda, 2002). Key aldehydes like hexanal, benzaldehyde, and (E)-2-hexenal, impart green and floral aromas, while saturated fatty aldehydes such as nonanal, hexanal, heptanal, and octanal contribute fruity, fatty, and green aroma. Hexanal was found as the key aroma contributor to the B2W9 and B2J9 samples with OAVs of 17 and 9.6 respectively (Table 3**)**. Elevated drying temperature decreases hexanal, with the highest level reported below 100 °C (Polat et al., 2018). Potentially the prolonged oxidation of amino acids followed by optimum drying assisted increase in hexanal in B2W9 and B2J9. Another aldehyde 3,6-Octadienal, 3,7-dimethyl- with floral-citrusy odor, and VIP >1, depicted high OAV ranging from 301.1 to 837.5 across WBT samples, compared to 33.5 to 50.1 in JBT samples, with particularly high OAVs in B2W3, B2W9, and B2J3. Our results highlight that 3,6-Octadienal, 3,7-dimethyl substantially contributes to the aroma of black tea. Moreover, heptanal attributed with grassy odor also revealed relatively high OAVs in B2W3 (4.4) and B2J3 (2.0), highlighting its potential role in the aroma of black tea. Previous studies have reported octanal and several other aldehydes as dominant flavor compounds (OAV >10) of fruity aroma in Fuyun-6 black tea (Huang et al., 2023). With fruity and floral aroma 2,6,6-trimethyl-1-cyclohexene-1-carboxaldehyde contribute to the sweet aroma in Fu brick tea. Our findings revealed that OAVs of 2,6,6-trimethyl-1-cyclohexene-1-carboxaldehyde gradually increased with fermentation time and subsequent drying at 95 °C, in following order B2W9 > B2W6 > B2W3. However, the level of this VOC remained rather stable.

The α-ionone and trans *β* ionone both carotenoid-derived VOCs impart floral and sweet aromas to black tea (Yu et al., 2021). These VOCs are generated via intrinsic asymmetric cleavage of *β* carotene employing *β*-carotene oxygenase, with high OAVs produce substantial floral and woody aromas of high grade Dianhong black tea (Ma et al., 2022). OAV of α-ionone depicted cultivar specific variation, with highest OAV (19.4) in B2W6 and 9.8 in B2J9. Similarly, trans *β* ionone also varied significantly ranging from 1073 in B2W6 to 304.5 in B2J6, denoting cultivar and processing conditions-specific variations in aroma formation. The *β* ionone is considered key aroma compound in black tea produced by the combined effect of fermentation followed by drying, as neither process can alone produce this compound. Its formation requires tea flavanol oxidation (fermentation of leaves for 1-3 h) followed by drying at 90 °C (Sanderson et al., 1971).

### 3.4 Analysis of VOCs detected by GC-IMS

**Fig. 2:**
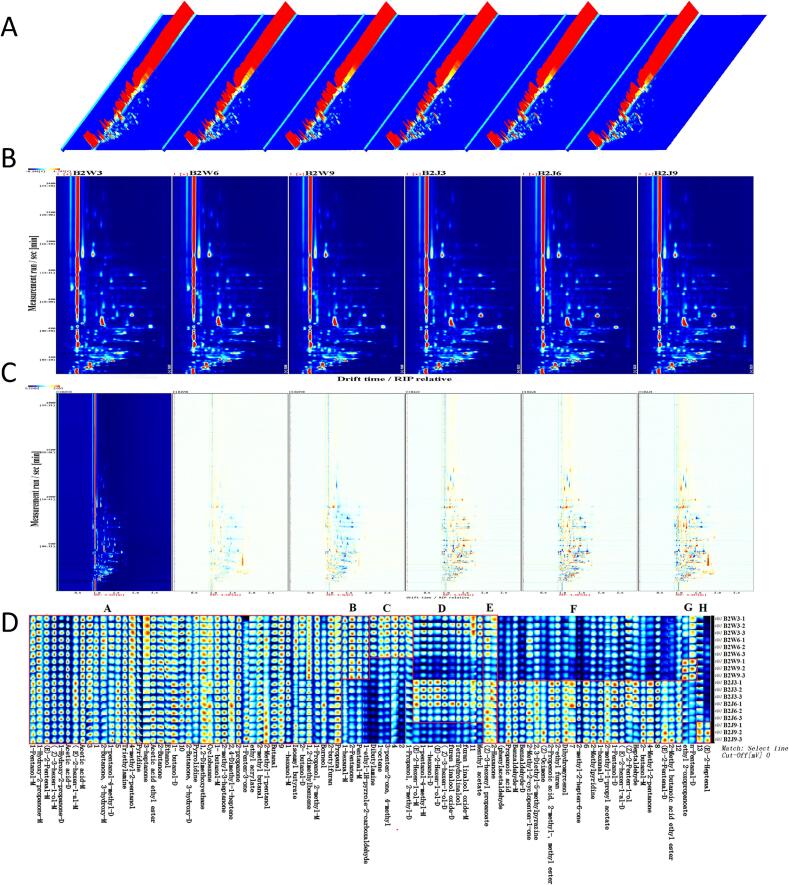
Spectrograms of VOCs in black tea samples of two cultivars with subjected to varying fermentation times followed by drying at 95 °C by using GC-IMS. (A) Three-dimensional topographic map of VOCs (B) Two-dimensional topographic map of VOCs (C) Two-dimensional topographic subtraction plot of VOCs showing difference comparison (D) Fingerprint plot of VOCs. The suffixes M and D represented monomer and dimer respectively.

Gas chromatography-ion mobility spectrometry exploits the differential mobility of compounds in an electric field to separate and detect ionized species under ambient pressure, offering an effective way to studying volatile compounds that define tea aroma. To further delineate volatile profile of black tea samples of both cultivars, VOCs were analyzed by GC-IMS, generating a three-dimensional spectrum (retention time, migration time, and peak intensity). The top view was taken to compare the differences. The background of the entire plot was blue, and the red vertical line at horizontal coordinate 1.0 was the RIP peak (reactive ion peak, normalized) (Fig. 2 **A)**. The vertical axis corresponds to the retention time (s) of the gas chromatogram while horizontal coordinate represent the ion migration time/drift time (normalized) (Fig. 2**B)**. To improve the discernibility of the differences among samples of black tea subjected to varying fermentation time followed by drying at 95 °C, a differential model was employed, using spectrum of B2W3 as reference. The spectra of other samples being derived by subtracting the reference spectrum of B2W3, resulted in white background color of the groups (Fig. 2**C)**. Each dot on either side of the RIP peak represents a VOC. The color denotes the concentration of the VOCs, with white and blue indicating a lower concentration, red and darkness intensity indicating a higher concentration. These figures elucidate the gas-phase ion mobility spectra of the six tea samples. The differences in VOCs among the six tea samples were obvious, with detected signals spanning retention time and drift time of 116–1569 s and 1–1.8 s respectively.

The VOCs fingerprints of six samples from the two cultivars were evaluated using all detected peaks (Fig. 2**D)**. Each line represents all the signal peaks selected in a sample whereas each column represents the signal peaks of the same VOC across samples (Fig. 2**D)**, with brighter color denoting higher concentration. The fingerprint plot depicted variations of VOCs between the samples of two cultivars. A total of 82 VOCs, excluding unidentified compounds were detected (Table 4**, and S6)** comprising 20 alcohol, 16 aldehydes, 14 ketones, 9 esters, 3 acids, 3 heterocyclic compounds, 2 furan, 1 ether, and 14 others. Certain VOCs including benzaldehyde, furan linalool oxide, (Z)-3-hexen-1-ol, (E)-2-Hexen-1-ol, 1-hexanol, 1-hydroxy-2-propanone, 2-butanone,3-hydroxy, 1-pentanol, (E)-2-hexen-1-al, (E)-2-pentenal, 1-propanol, 2-methyl, 1-hexanal, 2-butanol and pentanal showed multiple peak signals, suggesting monomeric and dimeric forms with varying concentrations across samples. Similar to the outcomes of the GC–MS shown in Fig. 1**C,** the numbers of VOCs with elevated abundance in B2W3 and B2J3 exceeded those in B2W6, B2W9 and B2J6, B2J9 respectively as shown in Fig. 2**D**. Moreover, the difference of the number of identified VOCs by GC–MS and GC-IMS could be attribute to the ability of the detection techniques and inherent composition of the two cultivars. However, detection of higher abundance/intensity of VOCs in B2W3 and B2J3 through both techniques underscore the directional changes produced by processing conditions despite inherent variations in metabolite composition of both cultivars. It suggests that processing conditions significantly altered the metabolites profile of both cultivars with best suitable conditions of 2.5 h fermentation time with subsequent drying temperature of 95 °C.

**Table 4:** VOCs identified using GC-IMS after varying fermentation time and drying temperatures.

	Compound	CAS#	Formula	MW	RI	Rt [*sec*]	Dt [a.u.]	Identification
1	Borneol	507–70-0	C_10_H_18_O	154.3	1734.5	1569.649	1.21498	RI, Dt
2	Phenylacetaldehyde	122–78-1	C_8_H_8_O	120.2	1646.1	1293.594	1.25232	RI, Dt
3	1-ethyl-1H-pyrrole-2-carboxaldehyde	2167–14–8	C_7_H_9_NO	123.2	1606.1	1185.36	1.17457	RI, Dt
4	Menthyl acetate	89–48-5	C_12_H_22_O_2_	198.3	1576.4	1110.761	1.22832	RI, Dt
5	Propanoic acid	79–09-4	C_3_H_6_O_2_	74.1	1574.3	1105.718	1.1039	RI, Dt
6	Benzaldehyde-M	100–52-7	C_7_H_6_O	106.1	1518	977.499	1.15664	RI, Dt
7	Benzaldehyde-D	100–52-7	C_7_H_6_O	106.1	1516.9	975.179	1.48007	RI, Dt
8	Acetic acid-M	64–19-7	C_2_H_4_O_2_	60.1	1483.8	906.978	1.05292	RI, Dt
9	Acetic acid-D	64–19-7	C_2_H_4_O_2_	60.1	1482.8	905.122	1.15218	RI, Dt
10	2,3-Diethyl-5-methylpyrazine	18,138–04-0	C_9_H_14_N_2_	150.2	1514.9	970.918	1.29533	RI, Dt
11	Dihydromyrcenol	53,219–21-9	C_10_H_20_O	156.3	1478.7	897.057	1.22687	RI, Dt
12	furan linalool oxide-M	60,047–17-8	C_10_H_18_O_2_	170.3	1470.3	880.644	1.2698	RI, Dt
13	furan linalool oxide-D	60,047–17-8	C_10_H_18_O_2_	170.3	1471.3	882.509	1.81742	RI, Dt
14	Tetrahydrolinalool	78–69-3	C_10_H_22_O	158.3	1445	833.268	1.264	RI, Dt
15	(*Z*)-3-hexen-1-ol-M	928–96-1	C_6_H_12_O	100.2	1404.8	763.137	1.22803	RI, Dt
16	(Z)-3-hexen-1-ol-D	928–96-1	C_6_H_12_O	100.2	1403.4	760.778	1.51305	RI, Dt
17	(E)-2-Hexen-1-ol-M	928–95-0	C_6_H_12_O	100.2	1427.2	801.446	1.3545	RI, Dt
18	(E)-2-Hexen-1-ol-D	928–95-0	C_6_H_12_O	100.2	1426.2	799.595	1.8171	RI, Dt
19	1-hexanol-M	111–27-3	C_6_H_14_O	102.2	1378.1	719.817	1.32439	RI, Dt
20	1-hexanol-D	111–27-3	C_6_H_14_O	102.2	1377.6	719.071	1.63661	RI, Dt
21	2-methyl-2-hepten-6-one	110–93-0	C_8_H_14_O	126.2	1359.2	690.739	1.18079	RI, Dt
22	1-pentanol-4-methyl-M	626–89-1	C_6_H_14_O	102.2	1347.8	673.59	1.63793	RI, Dt
23	1-pentanol-4-methyl-D	626–89-1	C_6_H_14_O	102.2	1347.2	672.845	1.32702	RI, Dt
24	(Z)-2-Penten-1-ol	1576-95-0	C_5_H_10_O	86.1	1346.7	672.099	1.45349	RI, Dt
25	1-Hydroxy-2-propanone-D	116–09-6	C_3_H_6_O_2_	74.1	1320.4	634.447	1.23349	RI, Dt
26	1-Hydroxy-2-propanone-M	116–09-6	C_3_H_6_O_2_	74.1	1320.5	634.642	1.03937	RI, Dt
27	(Z)-3-hexenyl propanoate	33,467–74-2	C_9_H_16_O_2_	156.2	1403.9	761.688	1.3719	RI, Dt
28	(E)-2-Heptenal	18,829–55-5	C_7_H_12_O	112.2	1343.9	667.987	1.24769	RI, Dt
29	2-Methyl-2-cyclopenten-1-one	1120-73-6	C_6_H_8_O	96.1	1378.7	720.731	1.42035	RI, Dt
30	2-Methyl-1-pentanol	105–30-6	C_6_H_14_O	102.2	1304	612.058	1.28419	RI, Dt
31	2-Butanone, 3-hydroxy-D	513–86-0	C_4_H_8_O_2_	88.1	1303.5	611.436	1.3347	RI, Dt
32	2-Butanone, 3-hydroxy-M	513–86-0	C_4_H_8_O_2_	88.1	1305.1	613.615	1.05688	RI, Dt
33	1-Pentanol-M	71–41-0	C_5_H_12_O	88.1	1266.5	546.367	1.25392	RI, Dt
34	2-Methylpyridine	109–06-8	C_6_H_7_N	93.1	1265.7	544.975	1.35004	RI, Dt
35	ethyl 2-oxopropanoate	617–35-6	C_5_H_8_O_3_	116.1	1270.2	553.05	1.43615	RI, Dt
36	1-Pentanol-D	71–41-0	C_5_H_12_O	88.1	1266.7	546.647	1.51088	RI, Dt
37	(E)-2-hexen-1-al-M	6728-26-3	C_6_H_10_O	98.1	1236.6	495.118	1.18669	RI, Dt
38	Isoamyl butyrate	2050-01-3	C_9_H_18_O_2_	158.2	1233.6	490.239	1.38231	RI, Dt
39	(E)-2-hexen-1-al-D	6728-26-3	C_6_H_10_O	98.1	1232.1	487.8	1.52187	RI, Dt
40	1,2-dimethylbenzene	95–47-6	C_8_H_10_	106.2	1218.3	466.096	1.07098	RI, Dt
41	Pyridine	110–86-1	C_5_H_5_N	79.1	1194.9	431.468	1.24351	RI, Dt
42	Heptaldehyde	111–71-7	C_7_H_14_O	114.2	1220	468.675	1.3423	RI, Dt
43	(E)-2-Pentenal-M	1576-87-0	C_5_H_8_O	84.1	1150.6	369.835	1.10904	RI, Dt
44	1- butanol-M	71–36-3	C_4_H_10_O	74.1	1159.1	380.919	1.18107	RI, Dt
45	2-Butylfuran	4466-24-4	C_8_H_12_O	124.2	1140.6	357.12	1.17733	RI, Dt
46	1- butanol-D	71–36-3	C_4_H_10_O	74.1	1157.1	378.311	1.38408	RI, Dt
47	3-penten-2-one, 4-methyl	141–79-7	C_6_H_10_O	98.1	1148.8	367.553	1.45144	RI, Dt
48	1-Propanol, 2-methyl-M	78–83-1	C_4_H_10_O	74.1	1107.9	318.65	1.17172	RI, Dt
49	1-hexanal-M	66–25-1	C_6_H_12_O	100.2	1097.3	306.995	1.26683	RI, Dt
50	1-hexanal-D	66–25-1	C_6_H_12_O	100.2	1099.8	309.658	1.5684	RI, Dt
51	1-Propanol, 2-methyl-D	78–83-1	C_4_H_10_O	74.1	1107.2	317.872	1.38824	RI, Dt
52	2-Hexanone	591–78-6	C_6_H_12_O	100.2	1103.4	313.66	1.48972	RI, Dt
53	Dibutylamine	111–92-2	C_8_H_19_N	129.2	1106.1	316.623	1.73133	RI, Dt
54	4-methyl-2-pentanol	108–11–2	C_6_H_14_O	102.2	1176.7	405.138	1.54355	RI, Dt
55	3-Heptanone	106–35-4	C_7_H_14_O	114.2	1172.4	399.026	1.58706	RI, Dt
56	1-Penten-3-one	1629-58-9	C_5_H_8_O	84.1	1061.9	274.11	1.08664	RI, Dt
57	Pyrrolidine	123–75-1	C_4_H_9_N	71.1	1059.9	272.441	1.04636	RI, Dt
58	2- butanol-M	78–92-2	C_4_H_10_O	74.1	1052.8	266.263	1.15061	RI, Dt
59	2-Pentanone	107–87-9	C_5_H_10_O	86.1	1027.9	245.893	1.11922	RI, Dt
60	Pentanal-M	110–62-3	C_5_H_10_O	86.1	1026.6	244.891	1.18749	RI, Dt
61	2-methyl-1-propyl acetate	110–19-0	C_6_H_12_O2	116.2	1024.9	243.542	1.24074	RI, Dt
62	2- butanol-D	78–92-2	C_4_H_10_O	74.1	1041.3	256.696	1.31961	RI, Dt
63	4-Methyl-2-pentanone	108–10-1	C_6_H_12_O	100.2	1019	238.988	1.4733	RI, Dt
64	n-Pentanal-D	110–62-3	C_5_H_10_O	86.1	1005.2	228.701	1.42881	RI, Dt
65	ethyl acrylate	140–88-5	C_5_H_8_O_2_	100.1	1002.4	226.677	1.39848	RI, Dt
66	2-Propenoic acid, 2-methyl-, methyl ester	80–62-6	C_5_H_8_O_2_	100.1	1002.2	226.508	1.36949	RI, Dt
67	2-Ethyl furan	3208-16-0	C_6_H_8_O	96.1	974.2	210.487	1.04931	RI, Dt
68	Ethanol	64–17-5	C_2_H_6_O	46.1	949.5	198.101	1.14526	RI, Dt
69	2-Butanone	78–93-3	C_4_H_8_O	72.1	951.9	199.23	1.2466	RI, Dt
70	1,2-Dimethoxyethane	110–71-4	C_4_H_10_O_2_	90.1	941.8	194.34	1.2985	RI, Dt
71	Butanal	123–72-8	C_4_H_8_O	72.1	910.3	179.858	1.29949	RI, Dt
72	2-methyl butanal	96–17-3	C_5_H_10_O	86.1	932.6	190.022	1.41507	RI, Dt
73	Acetic acid ethyl ester	141–78-6	C_4_H_8_O_2_	88.1	895.1	173.29	1.34319	RI, Dt
74	2-propanone	67–64-1	C_3_H_6_O	58.1	832.1	148.387	1.12415	RI, Dt
75	1-octene	111–66-0	C_8_H_16_	112.2	855.4	157.142	1.1628	RI, Dt
76	2,4-Dimethyl-1-heptene	19,549–87-2	C_9_H_18_	126.2	871.2	163.368	1.20688	RI, Dt
77	Propanal	123–38-6	C_3_H_6_O	58.1	801.7	137.687	1.14856	RI, Dt
78	Triethylamine	121–44–8	C_6_H_15_N	101.2	765	125.819	1.09227	RI, Dt
79	Cyclohexane	110–82-7	C_6_H_12_	84.2	734.3	116.675	1.14042	RI, Dt
80	(Z)-Ocimene	470–82-6	C_10_H_18_O	154.3	1213.2	458.28	1.29401	RI, Dt
81	(E)-2-Pentenal-D	1576-87-0	C_5_H_8_O	84.1	1143.3	360.576	1.36145	RI, Dt
82	2-Methyl butanoic acid ethyl ester	7452-79-1	C_7_H_14_O_2_	130.2	1058.7	271.357	1.23284	RI, Dt

Note: MW represented molecular weight; RI represented retention index; Rt represented retention time; Dt represented drift time.

#### 3.4.1 Analysis of VOCs elucidating cultivar dependent responses to processing conditions

To reveal combined effect of varying fermentation times followed by drying at 95 °C on VOCs profiles, and to evaluate how the processing induced variations were modulated by differently propagated tea cultivars, the fingerprint map was constructed. The comparison between clonally propagated Wanghai-1 and seed propagated JiuKeng cultivars was performed to exhibit cultivar specific responses to processing conditions, rather than to evaluate cultivar differences independently. We identified eight (A-G) changing trends in VOCs contents across all the samples of both cultivars (Fig. 2**D)**. The concentration of 36 VOCs in region (A) was significantly high, with some VOCs showing little or no change across all the samples. VOCs in region a mainly included (Z)-3-hexen-1-ol-M, 1-hexanol-M, (E)-2-hexen-1-al-M, 1-pentanol-M, 2-butanone, isoamyl butyrate, acetic acid ethyl ester, 2-butylfuran and several other VOCs were significantly high across samples of both cultivars. These VOCs were substantially retained across all the processing conditions, suggesting that these compounds likely contribute to the basic aroma profile of both cultivars. Regions **B, C, D, E, F, G,** and **H** elucidated differential volatile compounds (dVOCs) across all the samples. In region **B** we detected 1-hexanal-M, Pentanal-M with grassy odors, and 2-Pentanone having a sweet fruity, and woody aroma,1-ethyl-1H-pyrrole-2-carboxaldehyde with tea aroma notably high across all samples of WBT, but lowest in JBT as illustrated. Particularly, 2-Pentanone was exclusively high in B2W3. 2-Pentanone is a terpenoid-like aldehyde and ketone (KAT) category VOC produced through oxidative degradation of carotenes, implying that fermentation process play key role in the formation of this aroma compound in black tea (Sanderson et al., 1971). In region **C,** we detected high contents of dibutylamine,1-octene, 3-penten-2-one, 4-methyl, 1-Propanol and 2-methyl-D in B2W3 followed by B2W6, notably these VOCs were lowest across all samples of JBT. 1-Propanol has been identified as key aroma compound, particularly its dimer form is relatively high in black tea exceeding 20.0 μg/g (Guo et al., 2024). A total of 9 VOCs including (E)-2-hexen-1-ol-M contributing to the fresh aroma of high quality black tea, (*Z*)-3-hexen-1-ol-D imparting green notes, tetrahydrolinalool, and menthyl acetate with refreshing aroma were high in B2W3 and B2J3 as shown in region **D.** Gradual loss of these VOCs with prolonged fermentation in combination with drying at 95 °C suggest decrease in quality of black tea. dVOCs labeled in region **E** showed that 2-hexanone with fruity aroma depicted relatively high content in B2W3 and B2J3, similarly (z)-3-hexenyl propanoate was highly accumulated in B2W3 and B2J6 suggesting significant reduction with prolonged fermentation. Region **F** contained 22 VOCs including dihydromyrcenol, 2,3-diethyl-5-methylpyrazine, 2,3-Diethyl-5-methylpyrazine, 2-ethyl furan, and several other core VOCs with high abundance exclusively in JBT, notably the contents of 1-pentanol-D, heptaldehyde and benzaldehyde-M was high in B2J3. It suggested that prolonged fermentation caused gradual decline of these compounds. Moreover, these compounds were markedly low in WBT. Moreover, (Z)-2-penten-1-ol, 2-propenoic acid, 2-methyl-, methyl ester, dihydromyrcenol, and 2-methyl-2-hepten-6-one with fruity aroma showed elevated contents in JBT, 1-hexanal-D, (E)-2-hexen-1-al-D, (Z)-ocimene depicted exclusively high concentrations in samples B2J3, B2J6, and B2J9. This indicates that JBT samples exhibited greater diversity in the dVOCs with high abundances under given processing conditions, suggesting cultivar specific response to combined treatments of fermentation times and first drying temperature. These results aligns with the findings of GC–MS. VOCs in regions **G** and **H** were high in B2W9 and B2J9. A detailed comparative presentation of dVOCs between samples of both cultivars has been shown in **Fig. S1.**

#### 3.4.2 Principal component analysis (PCA) of tea samples of Wanghai and JiuKeng cultivars

PCA was performed to analyze the differentiation of black tea samples based on VOCs (**Fig. S2)**. PCA plot illustrated contribution of PC1 and PC2 of 47% and 28% respectively, with cumulative contribution of 75% to the total variance demonstrating good reliability of data. WBT was clearly segregated from JBT, with the first two principal components contributing 75% of total variance. B2W3, B2W6, and B2W9 clustered into one group, whereas B2J3, B2J6 and B2J9 clustered into another, indicating significant difference in aroma characteristics among samples of both cultivars subjected to combinations of varying fermentation time followed by drying at 95 °C.

#### 3.4.3 Screening of VOCs based on VIP and OAV values

**Fig. 3:**
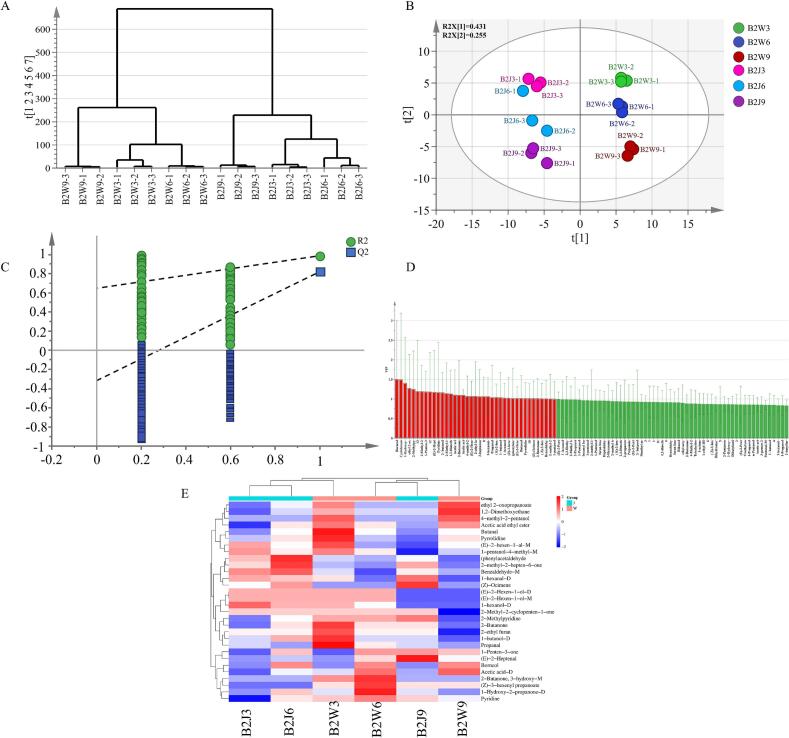
Multivariate analysis of black tea samples of WBT and JBT cultivars subjected to varying fermentation time and subsequent drying at 95 °C. (A) Hierarchical cluster analysis. (B) Scatter plot of scores of OPLS-DA (C) 200 permutation test validating model robustness. (D) VOCs highlighted in red depict VIP values higher than 1. (E) Heat map and hierarchical cluster of VOCs with VIP >1.

To compare the variation in VOCs of black tea samples due to combined effect of fermentation time followed by drying at 95 °C and to find the key differential VOCs, we employed OPLS-DA analysis. Hierarchical cluster analysis **(**Fig. 3 **A)** and score plot **(**Fig. 3**B)** depicted that model can effectively discern black tea samples subjected to the combinations of varying fermentation times followed by drying at 95 °C. The fitting parameters of the model, R^2^X = 0.945, R^2^Y = 0.969, Q^2^ = 0.855, suggest that model has high explanatory and predictive ability. Following 200 permutations the R^2^ regression line Y-axis intercept was 0.627 (R^2^ = (0.0, 0.627) and Q^2^ line Y-axis intercept was <0 (Q^2^ = 0.0, −0.323), suggesting that model was not overfitted and possesses high degree of validity. The hierarchical cluster analysis based on VOCs exhibited distinct grouping among black tea samples subjected to different fermentation time followed by drying at 95 °C (Fig. 3 A). The dendrogram had two primary clusters corresponding to the Wanghai and Jiukeng cultivars, denoting that cultivars also influenced composition of VOCs. Within each major cluster, samples of each cultivar were subdivided according to the processing conditions with clear grouping of B2W3, B2W6, and B2W9, as well as B2J3, B2J6, and B2J9. This clustering pattern suggest that while cultivar type orchestrate VOCs of black tea samples, the combined effect of fermentation time and first drying temperature of 95 °C significantly contribute to the VOCs profiles of samples from each cultivar. The score plot revealed distinct separation of two cultivars along the first predictive component t[1], elucidating that cultivar difference was also a source of variation in VOCs. Contrary to that, samples within each cultivar were separated along the second component t[2], making a gradient from B2W3/B2J3 to B2W9/B2J9. Interestingly, despite clear separation of two cultivars along first predictive component t[1], samples of each cultivar followed a comparable grouping along the second component where B2W3/B2J3, B2W6/B2J6, and B2W9/B2J9 were consistently grouped from negative to positive directions. The parallel distribution of samples along t[2] for both cultivars elucidate a conserved processing response, whereby combined effect of fermentation time followed by first drying at 95 °C modulates VOCs in a consistent directional manner across cultivars, despite inherent variations in their composition.

Notably, B2W3 and B2J3 samples were distinctly separated from other samples suggesting more similarity between these samples. From the perspective of the VOCs elucidated by the finger plots, it is consistent with the results of GC-IMS. The proximity of the replicates in score plot highlight consistency and reliability of the data.

To screen the key VOCs differentiating black tea samples due to processing parameters, variable importance in projection VIP values of each VOC was determined. To further screen key VOCs, we selected those VOCs with VIP > 1. Previous studies have reported that a compound with OAV < 1 is considered a non-significant contributor to the aroma (Yang et al., 2022), however, if its VIP > 1, it can potentially play a crucial role in the aroma formation and differentiation among tea samples (Zhang et al., 2024). A total of 35 VOCs were found with VIP > 1 excluding unidentified compounds **(**Fig. 3**D)**. VOCs with OAV >1 were 2,3-diethyl-5-methylpyrazine (coffee/nutty) and pyridine, contributing floral odor in B2J9 **Table S7**. Moreover, key dVOC 2-methyl butanoic acid ethyl ester also known as ethyl 2-methyl butyrate contributing to sweetness was particularly high in B2J6 and B2J9. In addition, the OAV values of phenylacetaldehyde (a product of L-Phenylalanine) with hyacinth aroma and hexanal, grassy aroma (Lu et al., 2024) were also high. Other VOCs with VIP > 1 made substantial contributions to distinguish aroma profiles of both cultivars at varying processing conditions.

The expression of VOCs with VIP > 1 (Fig. 3**E)** revealed significant variation among samples of both cultivars, with B2W3 and B2J3 exhibiting highest abundance of dVOCs. Particularly ethyl 2-oxopropanoate, 4-methyl-2-pentanol, butanal, 1-pentanol-4-methyl-M, 2-butanone, 2- ethyl furan, 1-butanol-D and propanal were significantly abundant in B2W3 indicates enzymatic transformation leading to aldehyde, alcohol and intermediate aroma compounds contributing to the aroma of B2W3 samples. However, these VOCs declined with prolonged fermentation in B2W6 and B2W9. The abundance of phenylacetaldehyde, 2-methyl 2-hepten-6-one, and benzaldehyde-M was relatively high in B2J6 and B2J3 whereas their expression substantially declined with prolonged fermentation as found in B2J9, indicating that prolonged fermentation accelerates their transformation or losses, thereby decreasing desirable aroma attributes. These findings clearly indicated that the expression of VOCs decreased as the fermentation time increased, moreover significant difference was observed among samples of same cultivar suggesting processing dependent variations. These results suggested that varying processing conditions altered the flavor profile, and that both cultivars have specific aroma compounds relative to the processing conditions. Our findings are in agreement with the previous reports that VOCs in tea first increase and then decrease as the fermentation time increases (Zheng et al., 2024).

### 3.5 Sensory evaluation

**Fig. 4:**
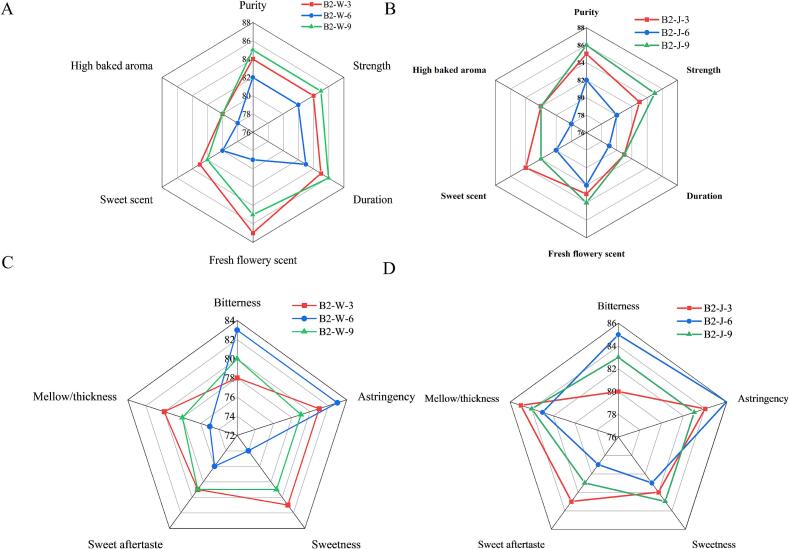
The Aroma and taste profiles of samples of WBT and JBT cultivars processed under varying fermentation time followed by drying at 95C°. (A, B) Taste profiles of samples of WBT and JBT cultivars. (C, D) aroma profiles of WBT and JBT cultivars.

**Fig. 5:**
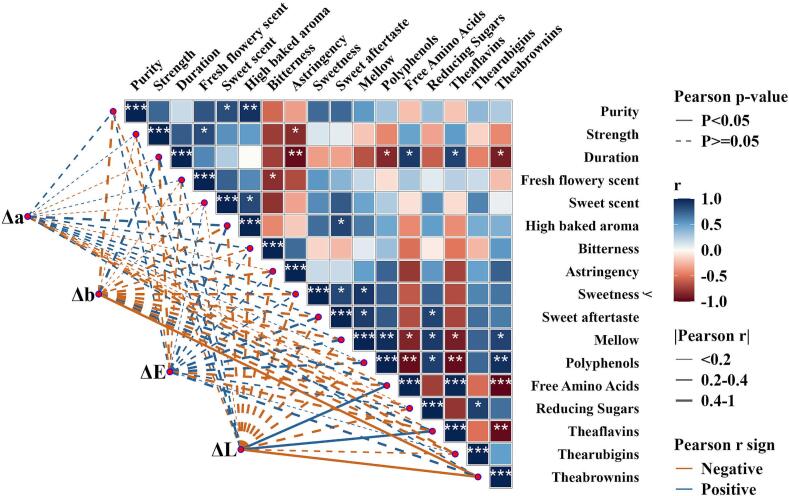
Network correlation of sensory and biochemical attributes. Blue color in heatmap highlight the positive correlation and red color depicts negative correlation. The asterisks denotes level of significance, “*” describes *p <* 0.05, “**” *p* < 0.01, “***” *p <* 0.001.

The sensory scores variations were well associated with the dynamic changes in key VOCs and biochemical components. In WBT, highest scores of aroma attributes including fresh flowery scent, sweet scent, and high baked aroma, and taste attributes like sweetness, sweet aftertaste, and mellow thickness of B2W3 (Fig. 4 **A)** corresponded with the higher accumulation of key aroma compounds such as linalool, cedrol and nonanal. Above mentioned sensory attributes were further enhanced with elevated amino acids and sugars. These findings elucidate that combined effect of fermentation time of 2.5 h followed by drying at 95 °C favored the retention of desirable flavor attributes. These findings were congruent with the biochemical analysis showing highest values of polyphenols, amino acids and reducing sugars in B2W3. Free amino acids contribute umami and sugar impart refreshing mellow sweetness and mellow attributes to the black tea infusions. (Long et al., 2024). Likewise, OAVs of cedrol (4.97), and nonanal (30.73) were highest in B2W3, contributing to woody and floral aroma. Notably, B2W3 contained the highest number of highly accumulated VOCs. Overall, these findings suggest that combined effect of fermentation treatment of 2.5 h followed by drying at 95 °C resulted more balanced aroma and taste, characterized by improved floral aroma and mellow thickness. In JBT, B2J3 had high scores of sweet scent, duration, high baked aroma, sweet aftertaste and mellow thickness whereas bitterness score was lowest, suggesting a more balanced flavor profile of B2J3. Linalool, with characteristics floral aroma exhibited highest OAV (8065) in B2J3 among all JBT samples. Similarly, OAVs of cedrol (1.88), nonanal (12.28) and trans-*β*-Ionone (406.7) were also high in B2J3, potentially contributing to the improved aroma profile. The network correlation analysis revealed association of sensory, biochemical and color attributes of tea infusion (Fig. 5). The detailed description of correlation has been given in supplementary file (**Fig. S3**).

## 4 Conclusion

This study evaluated the combined effect of different fermentation times and subsequent drying at 95 °C on the sensory attributes, biochemical composition, and volatile profiles of black tea employing GC–MS and GC-IMS. Multivariate analysis (PCA and OPLS-DA) demonstrated that processing conditions had significantly influenced evolution of VOCs, with 2.5 h fermentation and subsequent drying at 95 °C was best among the tested combinations of the processing conditions yielding optimal quality. A total of 98 and 82 VOCs, including terpene alcohol, aldehydes, ketones and esters were identified based on GC–MS and GC-IMS, respectively. The variations in the composition and concentration of these VOCs potentially changed the flavor characteristics of black tea. Key differential VOCs including linalool, 1-nonanol, cedrol, nonanal, 2,3-diethyl-5-methylpyrazine, 3,6-octadienal, 3,7-dimethyl-, and ethyl 2-methylbutyrate significantly contributed to the aroma attributes.

Our study underscores the pertinent role of combined effect of fermentation times and subsequent drying conditions in modulating black tea quality, and provide practical basis for process optimization in industrial production. Prospective studies should include tea samples collected across multiple seasons and location, and broader range of processing parameters to confirm general applicability of these findings.

## CRediT authorship contribution statement

**Muhammad Yasir:** Writing – original draft, Software, Methodology, Investigation, Data curation, Conceptualization. **Yanhua Jiang:** Data curation. **Noman Walayat:** Writing – review & editing. **Jameel M. Al-Khayri:** Writing – review & editing, Supervision, Funding acquisition. **Mohammed I. Aldaej:** Validation, Formal analysis. **Muneera Q. Al-Mssallem:** Writing – review & editing, Visualization. **Fatima M. Alessa:** Writing – review & editing. **Zhucheng Su:** Project administration. **Mustafa I. Almaghasla:** Software. **Ran Wei:** Supervision.

## Funding sources

This work was supported by Major Special Project for Agricultural Technology Extension in Zhejiang Province (2024ZDXT05–09), National Natural Science Foundation of China (32302609), and Ningbo Tea Industry Technology Team Project (2024-2025).

This work was supported by the Deanship of Scientific Research, Vice Presidency for Graduate Studies and Scientific Research, King Faisal University, Saudi Arabia [Grant No. KFU262648].

## Declaration of competing interest

The authors declare that they have no known competing financial interests or personal relationships that could have appeared to influence the work reported in this paper.

## Data Availability

No data was used for the research described in the article.
