# Supplementary material for: Exploring the combined effect of fermentation time, and first drying temperature on black tea flavor profile characterized by GC–MS and GC-IMS
Source: Food Chem X. 2026 May 12;36:103975. doi: 10.1016/j.fochx.2026.103975 (PMC13208830; doi:10.1016/j.fochx.2026.103975)
Supplement: Supplementary file 1 — Supplementary material 1 [file mmc1.docx]

Supplementary figures


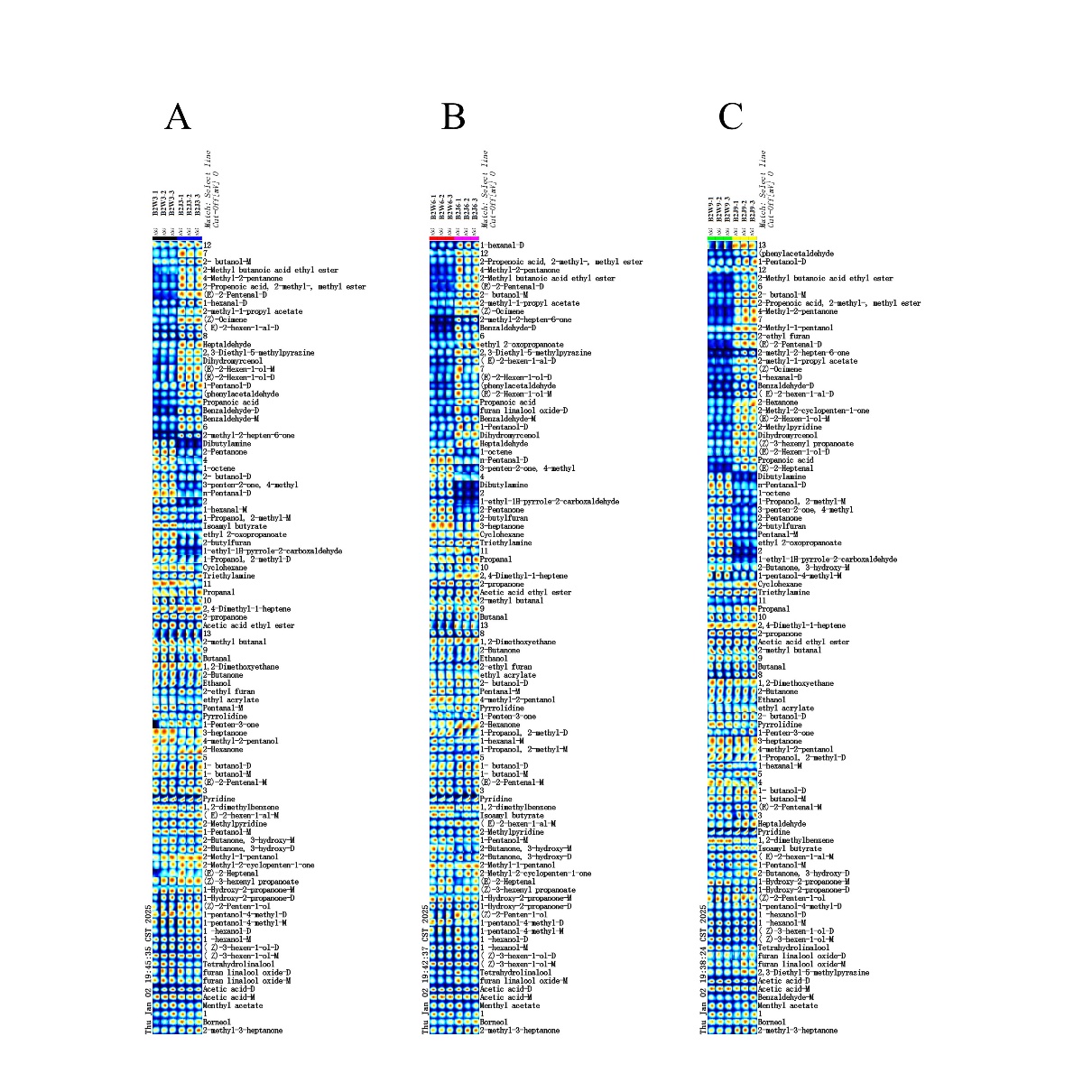


**Figure S1**. Fingerprint plot of VOCs across the samples subjected to varying feremenntation times (2.5h, 4h and 5.5h) followed by first drying temperature of 95 ℃. (A) finger print plot comparing Wanghai black tea samples (B2W3; 2.5h ferementation, 95 ℃ first drying) and Jiukeng black tea (B2J3; 2.5h ferementation, 95 ℃ first drying) (B) finger print plot comparing Wanghai black tea samples (B2W6; 4h ferementation, 95 ℃ first drying) and Jiukeng black tea (B2J6; 4h ferementation, 95 ℃ first drying) (C) finger print plot comparing Wanghai black tea samples (B2W9; 5.5h ferementation, 95 ℃ first drying) and Jiukeng black tea (B2J9; 5.5h ferementation, 95 ℃ first drying). The numbers in the fingerprint plot denotes unidentified compounds in the refernce library.





**Figure S2.** Principal component analysis (PCA) score plot illustrating the combined effect of varying times (2.5, 4 and 5.5h) followed by first drying at 95 ℃ and qualitative spectra of VOCs detected by GC-IMS (A) PC1 (47%) and PC2 (28%) explain 75% of total variance. Samples distribution indicates distinct separation driven by processing conditions. Replicates close location, confirms significant reproducibility. (B) Two dimensional spectra of VOCs denotes the qualitative VOCs profile. The x-axis denotes drift time (relative to the reaction ion peak, RIP) whereas y-axis represent retention time. Color gradient blue to red shows signal intensity. Each spot corresponds to individual VOC or its monomer/dimer form. Dense distribution and variation in intensities of signals depicts complexity of VOCs composition and their dynamic modulation due to combined effect of fermentation durations and first drying temperature, highlighting strong impact of processing conditions on aroma profile.

**Correlation network analysis of biochemical, taste and color attributes of black tea**

In current study we explored the correlation among biochemical, sensory and color attributes of black tea infusions. The findings of the color difference analysis revealed that the ΔL values were negative (Table S2), suggesting that infusion color was darker than the water. The ΔL values were highly negatively and significantly correlated with TB contents **(Fig. S3)**. It highlights that prolonged fermentation caused higher accumulation of TB contents leading to the darker infusion color and reduced brightness with inferior sensory quality (Hua et al., 2022). Previous studies have reported similar trend in black tea (Jua, 2014; Su et al., 2024) In contrast ΔL values were highly positively and significantly correlated with theaflavin and amino acid contents. TF are produced through oxidative conversion of catechins during fermentation thereby improving brightness of the tea infusions. During fermentation TF contents increase initially but then decrease. Prolonged fermentation was unfavorable for the infusion brightness and resulted in the accelerated formation of TR and TB (Hua et al., 2022). Similarly amino acids tend to increase up to certain extent during fermentation but decrease after 6 hours of fermentation. The initial increase could be attributed to the degradation of proteins by peptidase and protease enzymes whereas decrease in free amino acid could be due to reaction with orthoquinones (Aaqil et al., 2024; Zhao et al., 2023). The Δb values of black tea infusions were on the positive scales (Table S2), suggesting that the black tea infusions were red and yellow in appearance. The Δb values were negatively and significantly associated with TB contents, suggesting that increase in TB contents decreased the redness of the infusions thereby decreasing the quality of the black tea. In addition, Δb was negatively correlated with most of the biochemical, taste and aroma attributes but the association was significantly weak. Our findings revealed that ΔE was negatively correlated with free amino acids and theaflavin contents but the association was non-significant. The Δa was positively correlated with most of the biochemical, taste and aroma attributes, suggesting that improved redness of the black tea infusion indirectly underscores the biochemical transformation during fermentation.

The association of flavor and biochemical attributes illustrated that the purity had significant positive correlation with high baked aroma (*p* < 0.01) and sweet scent (*p* < 0.05), whereas duration attribute had significant positive correlation with TF and free amino acid contents. Our results demonstrated that B2W3, B2W9, B2J3 and B2J9 had highest values of aroma duration **(Fig. 4).** These results coincides with the elevated values of free amino acids in in B2W3 and B2W9 and further supported by high values of TF in B2W3. These results suggest that persistence of aroma duration during at early stage of fermentation was in association with combined effect of high TF and free amino acids, whereas later stage persistence was due to amino acids. The high baked aroma and had significant positive association with sweet-aftertaste (*p* < 0.05). Whereas, sweetness had significant positive association with mellow and sweet-aftertaste sensory scores (*p* < 0.05). Mellowness was highly positively and significantly correlated with polyphenols contents (*p* < 0.01). Our findings revealed that B2W3 and B2J3 had significantly high polyphenol contents (Table 1). A prior study explained that interaction of polysaccharide conjugates and TB improved the taste characteristics including mellowness in Pue-erh tea (Deng et al., 2024). However, another study the inverse association of polyphenols and mellow/thickness of black tea infusions (Chen et al., 2024). TF contents were also highly positively and significantly correlated with amino acid contents (*p* < 0.001). Oure results reported TF and free amino acids were notably retained at shorter fermentation duration, suggesting improved taste of black tea infusions. Previous studies reported that TF and free amino acids are significantly and positively correlated with the quality scores of black tea (Liang et al., 2003). In contrast TB contents showed highly negative and significant correlation with amino acid contents (*p* < 0.001), suggesting that as the TB accumulate, retention of free amino acids declines. TF also showed highly negative and significant correlation with TB (*p* < 0.01). Above mentioned results explored that black tea quality was significantly mediated by combined effect of fermentation time followed by first drying temperature driven biochemical variations. Shorter duration of fermentation (B2W3/B2J3) with higher TF and free amino acids improved brightness, duration, mellow/thickness, and sweet aftertaste. Prolonged fermentation in B2W5 and B2J9 led to increased TB contents with dark infusion color (lower ΔL), high astringency and strength. Color attributes particularly ΔL and Δb clearly exhibited the biochemical variations, like strong inverse correlation between ΔL and TB and TF-TB. Sensory attributes were primarily mediated by TF and free amino acids interaction at early fermentation and free amino acids and TB at prolonged fermentation.





**Figure S3.** A detailed network correlation of sensory attributes with other key quality parameters of black tea quality (A) Network correlation of sensory and biochemical and infusion color attributes. Blue color in heatmap highlight the positive correlation and red color depicts negative correlation. (B) Network correlation of sensory (taste and aroma) and biochemical attributes. The asterisks denotes level of significance, “*” describes *p <* 0.05, “**” *p* < 0.01, “***” *p <* 0.001. The network exhibit correlation of all attributes among each other.

References

Aaqil, M., Kamil, M., Kamal, A., Nawaz, T., Peng, C., Alaraidh, I. A., Al-Amri, S. S., Okla, M. K., Hou, Y., Fahad, S., & Gong, J. (2024). Metabolomics reveals a differential attitude in phytochemical profile of black tea (Camellia Sinensis Var. assamica) during processing. *Food Chemistry: X*, *24*, 101899. <https://doi.org/https://doi.org/10.1016/j.fochx.2024.101899>

Chen, Q., Fu, Y., Heng, W., Yu, S., Xie, F., Dong, F., Lin, Z., Dai, W., & Fu, H. (2024). Re-rolling treatment in the fermentation process improves the taste and liquor color qualities of black tea. *Food Chemistry: X*, *21*, 101143. <https://doi.org/https://doi.org/10.1016/j.fochx.2024.101143>

Deng, S., Zhang, T., Fan, S., Na, H., Dong, H., Wang, B., Gao, Y., Xu, Y.-Q., & Liu, X. (2024). Polysaccharide Conjugates' contribution to mellow and thick taste of Pu-erh ripe tea, besides Theabrownin. *Food Chemistry: X*, *23*, 101726. <https://doi.org/https://doi.org/10.1016/j.fochx.2024.101726>

Hua, J., Wang, H., Yuan, H., Yin, P., Wang, J., Guo, G., & Jiang, Y. (2022). New insights into the effect of fermentation temperature and duration on catechins conversion and formation of tea pigments and theasinensins in black tea. *Journal of the Science of Food and Agriculture*, *102*(7), 2750-2760. <https://doi.org/https://doi.org/10.1002/jsfa.11616>

Jua, Y. (2014). Study on Correlations between Color and Quality of Congou Made of Different Varieties of Tea. *Southwest China Journal of Agricultural Sciences*.

Liang, Y., Lu, J., Zhang, L., Wu, S., & Wu, Y. (2003). Estimation of black tea quality by analysis of chemical composition and colour difference of tea infusions. *Food Chemistry*, *80*(2), 283-290. <https://doi.org/https://doi.org/10.1016/S0308-8146(02)00415-6>

Su, S., Long, P., Zhang, Q., Wen, M., Han, Z., Zhou, F., Ke, J., Wan, X., Ho, C.-T., & Zhang, L. (2024). Chemical, sensory and biological variations of black tea under different drying temperatures. *Food Chemistry*, *446*, 138827. <https://doi.org/https://doi.org/10.1016/j.foodchem.2024.138827>

Zhao, F., Wu, W., Wang, C., Wang, X., Liu, H., Qian, J., Cai, C., Xie, Y., & Lin, Y. (2023). Dynamic change of oligopeptides and free amino acids composition in five types of tea with different fermentation degree processed from the same batch of fresh tea (Camelilia Sinensis. L.) leaves. *Food Chemistry*, *404*, 134608. <https://doi.org/https://doi.org/10.1016/j.foodchem.2022.134608>
