# Supplementary material for: Exploring the combined effect of fermentation time, and first drying temperature on black tea flavor profile characterized by GC–MS and GC-IMS
Source: Food Chem X. 2026 May 12;36:103975. doi: 10.1016/j.fochx.2026.103975 (PMC13208830; doi:10.1016/j.fochx.2026.103975)
Supplement: Supplementary file 2 — Supplementary material 2 [file mmc2.docx]

**Table S1**. Sample names and processing conditions

| Sample names | Fermentation times | Drying temperatures |
| --- | --- | --- |
| B2W1 | 2.5h | 125℃ |
| B2W2 |  | 110℃ |
| B2W3 |  | 95℃ |
| B2W4 | 4h | 125℃ |
| B2W5 |  | 110℃ |
| B2W6 |  | 95℃ |
| B2W7 | 5.5h | 125℃ |
| B2W8 |  | 110℃ |
| B2W9 |  | 95℃ |
| B2J1 | 2.5h | 125℃ |
| B2J2 |  | 110℃ |
| B2J3 |  | 95℃ |
| B2J4 | 4h | 125℃ |
| B2J5 |  | 110℃ |
| B2J6 |  | 95℃ |
| B2J7 | 5.5h | 125℃ |
| B2J8 |  | 110℃ |
| B2J9 |  | 95℃ |

Note: BW corresponds to the black tea samples derived from Wanghai cultivar, whereas BJ corresponds to the black tea samples derived from Jiukeng cultivar, 2 stands for the two leaves and one bud stage of tea leaves, whereas numbers 1-9 denotes different processing conditions (combinations of fermentation time and first drying temperature).

**Table S2.** Infusion color difference indicator of black tea samples processed with varying fermentation times and drying temperatures (mean ± SD^a^)

| Samples | ΔE | ΔL | Δa | Δb |
| --- | --- | --- | --- | --- |
| B2W1 | 4.97±0.40d | -3.60±0.56bc | 1.03±0.38bc | 3.13±0.99c |
| B2W2 | 4.77±0.49d | -3.17±0.55abc | 0.90±0.61c | 3.23±1.24bc |
| B2W3 | 5.40±0.82bcd | -2.07±0.50a | 1.50±0.44abc | 4.70±0.78ab |
| B2W4 | 5.27±0.57cd | -2.97±0.25abc | 1.27±0.31abc | 4.17±0.65abc |
| B2W5 | 6.27±0.06ab | -3.60±0.96bc | 1.47±0.32abc | 4.83±0.72a |
| B2W6 | 6.03±0.15abc | -3.00±0.17abc | 1.70±0.10ab | 4.97±0.21a |
| B2W7 | 6.37±0.25ab | -3.53±0.25bc | 1.90±0.10a | 4.90±0.36a |
| B2W8 | 6.63±0.45a | -4.17±0.80c | 1.77±0.49a | 4.70±1.13ab |
| B2W9 | 6.37±0.92ab | -2.80±1.21ab | 2.00±0.36a | 5.33±0.38a |
| B2J1 | 6.53±0.68ab | -4.60±0.69ns | 1.97±0.23ab | 4.17±0.25bc |
| B2J2 | 6.03 ±0.50b | -4.33±1.67ns | 1.30±0.69c | 3.53±1.42c |
| B2J3 | 6.67±0.31ab | -4.43±0.47ns | 1.77±0.15abc | 4.60±0.36abc |
| B2J4 | 6.53±0.15ab | -4.03±0.06ns | 2.03±0.15ab | 4.70±0.20ab |
| B2J5 | 7.07±0.46a | -4.30±0.26ns | 1.90±0.35ab | 5.27±0.29a |
| B2J6 | 6.23±0.38ab | -3.73±0.38ns | 1.73±0.12bc | 4.70±0.17ab |
| B2J7 | 6.57±0.31ab | -4.37±0.40ns | 2.33±0.23a | 4.27±0.15abc |
| B2J8 | 6.17±0.67ab | -3.83±0.71ns | 2.10±0.10ab | 4.37 0.31abc |
| B2J9 | 6.8±0.61ab | -5.10±1.22ns | 2.07±0.15ab | 3.93±0.57bc |

Different lower-case letters indicate significant differences among samples of each cultivar (*p* < 0.05). The data was shown as the mean values ± standard deviation.

**Table S3.** VOCs detected by GC-MS

|  |  |  |  |  |  | Content µg/g | | | | | |
| --- | --- | --- | --- | --- | --- | --- | --- | --- | --- | --- | --- |
| # | Compounds | Odor description | CAS | Category | VIP values | B2W3 | B2W6 | B2W9 | B2J3 | B2J6 | B2J9 |
| 1 | Furan, 2-ethyl- | Smoky burnt aroma | 3208-16-0 | Furan | 0.710935 | 5.76±  0.69c | 3.57± 0.49d | 10.74± 0.42a | 10.55± 0.69a | 7.79± 0.47b | 10.73±  0.63a |
| 2 | Hexanal | Grassy, green, fresh, fatty | 66-25-1 | Aldehyde | 1.31126 | 68.00±  3b | 67.74± 6.89b | 76.00±  1.00a | 39.00±  1.00c | 27.12±  2.01d | 42.67±  3.79c |
| 3 | 3-Furaldehyde | Sweet, bready, caramel | 498-60-2 | Aldehyde | 0.19085 | 14.3±  0.61a | 2.54±  0.23d | 3.75±  0.1cd | 4.66±  0.59c | 6.62±  1.99b | 8.11±  0.57b |
| 4 | 3-Methylbut-2-enoic acid, 4-nitrophenyl ester | nd | 1000307-59-8 | Acid | 0.757124 | 88.48±  1.06e | 123.00±  1.00b | 132.10±  1.06a | 90.69±  1.64d | 85.04±  0.95f | 99.31±  1.03c |
| 5 | Oxalic acid, allyl butyl ester | nd | 1000309-23-1 | Acid | 0.837683 | 117.80±  1.25c | 150.60±  1.16b | 176.70±  1.47a | 114.67±  0.58d | 112.55±  2.33d | 117.60±  2.39c |
| 6 | Propanoic acid, anhydride | Pungent odor | 123-62-6 | Acid | 0.796329 | 109.10±  1.52d | 138.90±  1.65b | 161.10±  1.60a | 107.69±  0.60d | 101.09±  0.58e | 115.12±  0.60c |
| 7 | 2-Propenal | Green, herbal | 107-02-8 | Aldehyde | 0.937966 | 58.01±  0.10b | 46.28±  1.50c | 71.06±  1.05a | 43.53±  0.89d | 39.21±  0.54e | 38.41±  0.53e |
| 8 | Heptanal | Green, oily, grassy | 111-71-7 | Aldehyde | 1.24943 | 27.15±  0.79a | 17.86±  1.30c | 20.14±  0.30b | 12.13±  1.33d | 10.89±  1.01d | 12.21±  0.57d |
| 9 | 2-Heptenal, (E)- | Fatty, oily, green, creamy | 18829-55-5 | Aldehyde | 0.838021 | 4.45±  0.52a | 2.55±  0.50a | 4.00±  2.34a | 2.30±  0.50a | 2.36±  0.48a | 3.88±  1.37a |
| 10 | Benzaldehyde | Almond-like, fruity, cherry-like, powdery, nutty | 100-52-7 | Aldehyde | 0.027949 | 140.00±  1.49d | 123.50±  0.62f | 126.80±  0.28e | 153.00±  0.16b | 144.95±  0.84c | 170.40±  0.53a |
| 11 | 1-Heptanol | Green, sweet, leafy | 111-70-6 | Alcohol | 1.25939 | 18.26±  0.68a | 12.63±  0.38b | 13.30±  0.61b | 9.00±  0.01c | 4.32±  0.17d | 3.51±  0.50d |
| 12 | 1-Octen-3-ol | Earthy, green, oily, vegetative-like, fungal | 3391-86-4 | Alcohol | 0.596876 | 25.07±  0.25a | 22.97±  1.76b | 25.05±  0.24a | 25.21±  0.69a | 19.07±  0.88c | 24.53±  0.50ab |
| 13 | 5-Hepten-2-one, 6-methyl- | Citrus-like, fruity odor | 110-93-0 | Ketone | 0.254126 | 10.16±  0.55d | 10.22±  0.48d | 12.70±  0.39c | 14.15±  0.30b | 13.03±  0.19c | 16.02±  0.97a |
| 14 | .beta.-Myrcene | Woody, resinous, musty | 123-35-3 | Terpene | 0.092263 | 60.46±  1.53c | 56.68±  1.50d | 43.12±  1.73f | 72.85±  2.19a | 53.27±  1.66e | 68.75±  0.66b |
| 15 | 4-Benzyloxy-N-methylamphetamine | nd | 177079-66-2 | Phenylpropanoid | 0.731599 | 10.72±  0.95c | 12.75±  0.56b | 8.89±  0.41d | 16.96±  0.82a | 13.26±  0.82b | 16.45±  1.06a |
| 16 | Hexanoic acid, ethyl ester | Fruity odor | 123-66-0 | Acid | 0.186712 | 3.41±  0.23b | 4.74±  0.06ab | 5.56±  0.51a | 6.07±  0.97a | 5.78±  1.73a | 5.79±  1.81a |
| 17 | Octanal | Citrus note | 124-13-0 | Aldehyde | 1.16874 | 7.57±  0.51a | 4.35±  0.44bc | 4.67±  0.58b | 2.69±  0.53d | 2.27±  0.31d | 3.64±  0.2c |
| 18 | 2,4-Heptadienal, (E,E)- | Fatty, green aroma. | 3/5/4313 | Aldehyde | 1.0059 | 1.42±  0.49d | 2.52±  0.33bc | 1.8±  0.77cd | 4.99±  0.79a | 3.37±  0.65b | 5.91±  0.08a |
| 19 | Ethanone,  1-(3-hydroxyphenyl)- | nd | 121-71-1 | Ketone | 0.35615 | 1.25±  0.32b | 1.85±  0.65ab | 2.38±  0.37a | 2.17±  0.29a | 1.28±  0.06b | 1.93±  0.74ab |
| 20 | o-Cymene | nd | 527-84-4 | Terpene | 0.060875 | 2.44±  0.58a | 2.27±  0.82a | 1.78±  0.75a | 2.5±  0.28a | 2.46±  0.44a | 2.63±  0.97a |
| 21 | D-Limonene | Citrus, lemon, orange-like, green | 5989-27-5 | Terpene | 0.690225 | 10.5±  0.5a | 8.11±  0.58b | 7.73±  0.52b | 8.65±  1.1b | 7.37±  0.65b | 8.56±  1.77b |
| 22 | 1-Hexanol, 2-ethyl- | Green, grassy | 104-76-7 | Alcohol | 0.513041 | 21.53±  0.50a | 15.76±  0.51d | 19.4±  1.03bc | 20.7±  1.49ab | 17.97±  0.9c | 18.64±  0.71c |
| 23 | Benzyl alcohol | Sweet, floral, rose-like, caramel | 100-51-6 | Alcohol | 0.372958 | 52.5±  0.51c | 32.53±  1.13e | 37.41±  0.52d | 60.04±  1.00c | 53.06±  0.14b | 68.16±  0.95a |
| 24 | 3-Octen-2-one |  | 1669-44-9 | Ketone | 0.174022 | 2.87±  0.10a | 2.97±  1.64a | 2.81±  0.17a | 4.50±  0.64a | 2.78±  0.44a | 3.99±  1.29a |
| 25 | Benzeneacetaldehyde | Floral, rose, cherry-like | 122-78-1 | Aldehyde | 0.041656 | 762.70±  2.18d | 688.15±  2.00e | 879.40±  0.59b | 879.10±  1.86b | 870.46±  2.18c | 1001.70±  1.5a |
| 26 | Nonane,  5-(2-methylpropyl)- | nd | 62185-53-9 | Hydrocarbon | 0.504725 | 19.74±  0.17b | 23.29±  0.53a | 7.70±  0.75c | 3.27±  0.82d | 8.00±  0.01c | 2.39±  0.81d |
| 27 | Nonane, 4,5-dimethyl- | nd | 17302-23-7 | Hydrocarbon | 1.01335 | 53.13±  0.99c | 43.52±  0.93b | 69.36±  0.10a | 30.26±  0.04d | 19.09±  0.06f | 25.31±  0.19e |
| 28 | 2-Butenal | Pungent | 4170-30-3 | Aldehyde | 0.847127 | 11.07±  0.07b | 7.60±  0.56d | 13.18±  0.73a | 7.11±  0.77d | 5.23±  0.66e | 9.47±  0.10c |
| 29 | Acetophenone | Sweet, cherry-like, vanilla-like | 98-86-2 | Ketone | 1.26312 | 14.70±  0.39a | 6.93±  1.45b | 6.49±  0.06b | 3.10±  0.29c | 3.19±  0.41c | 3.59±  0.24c |
| 30 | 2-Furanmethanol,  5-ethenyltetrahydro-  .alpha.,.alpha.,5-trimethyl-,  cis- | Earthy floral sweet woody | 5989-33-3 | Furan | 1.13514 | 113.28±  0.94b | 111.41±  1.1b | 137.1±  0.18a | 82.16±  1.93c | 72.12±  1.14d | 73.51±  0.9d |
| 31 | 1-Propanone,  1-(2-furanyl)- |  | 3194-15-8 | Ketone | 0.596356 | 15.22±  0.83d | 14.33±  0.58d | 6.63±  0.44e | 30.42±  0.52a | 16.89±  0.87c | 25.69±  1.23b |
| 32 | Formic acid, octyl ester | Fruity | 112-32-3 | Acid | 1.3414 | 29.24±  1.06a | 25.71±  0.62a | 27.08±  1.06a | 15.26±  2.61b | 12.02±  2.08b | 14.7±  3.67b |
| 33 | 1,3-Cyclohexadiene,  1-methyl-4-(1-methylethyl)- | Lemon-citrus | 99-86-5 | Terpene | 0.487788 | 1.91±  0.77a | 0.99±  0.21a | 1.22±  0.69a | 1.45±  0.22a | 1.16±  0.08a | 1.44±  0.58a |
| 34 | trans-Linalool oxide (furanoid) | Floral type odor | 34995-77-2 | Terpene | 0.960806 | 185.16±  1.00c | 179.3±  0.85b | 220.6±  0.66a | 155.7±  0.94d | 139.48±  0.51e | 139±  0.89e |
| 35 | Undecane, 5,7-dimethyl- | nd | 17312-83-3 | Hydrocarbon | 1.41102 | 1053.00±  2.30b | 1023.20±  2.60c | 1179.08±  1.00a | 503.1±  1.83d | 29.19±  1.18e | 29.13±  0.13e |
| 36 | Linalool | Floral, sweet, grape-like, woody | 78-70-6 | Terpene Alcohol | 1.31554 | 1073.70±  0.40b | 1025.4±  0.50c | 1175.7±  0.74a | 701.85±  2.67d | 507.29±  0.42e | 472.1±  1.02f |
| 37 | 1,5,7-Octatrien-3-ol,  3,7-dimethyl- | nd | 29957-43-5 | Terpene Alcohol | 0.587889 | 75.07±  0.94b | 77.47±  0.57b | 621.30±  1.35a | 67.12±  1.99c | 51.73±  2.58e | 56.06±  2.68d |
| 38 | Nonanal | Floral, fatty, green, lemon-like | 124-19-6 | Aldehyde | 1.37287 | 87.09±  0.94a | 62.78±  0.70b | 62.44±  1.41b | 34.77±  1.31c | 25.98±  1.71e | 32.02±  1.84d |
| 39 | Phenylethyl Alcohol | Floral, rose-like | 12/8/1960 | Alcohol | 0.85217 | 139.7±  0.54d | 100.8±  1.17f | 111.0±  0.94e | 241.09±  0.94b | 198.06±  2.10c | 249.7±  2.46a |
| 40 | Undecane, 4-methyl- | nd | 2980-69-0 | Hydrocarbon | 0.712644 | 58.8±  1.51c | 69.0±  0.06ba | 70.38±  0.23a | 67.42±  1.45b | 51.34±  2.17d | 52.25±  1.80d |
| 41 | (R,S)-5-Ethyl-6-methyl-3E-hepten-2-one | fresh | 57283-79-1 | Ketone | 0.374091 | 5.02±  0.95c | 6.08±  0.07b | 5.19±  0.04bc | 9.70±  0.62a | 6.05±  0.61b | 9.24±  0.25a |
| 42 | 1,6-Heptadiene, 3-methyl- | nd | 50871-05-1 | Hydrocarbon | 1.0021 | 5.01±  0.01ab | 4.78±  0.10abc | 5.38±  0.65a | 4.33±  0.34abc | 3.51±  0.57c | 3.85±  1.50bc |
| 43 | 2-Pentadecyn-1-ol | nd | 2834-00-6 | Alcohol | 0.634102 | 1.87±  1.28a | 1.49±  0.46a | 1.10±  0.47a | 1.33±  0.39a | 1.1±  0.27a | 1.7±  0.47a |
| 44 | (3R,6S)-2,2,6-Trimethyl-6-vinyltetrahydro-2H-pyran-3-ol | Earthy or woody aromas | 39028-58-5 | Terpene Alcohol | 1.26395 | 14.16±  0.74a | 12.40±  1.37ab | 13.91±  1.07a | 9.05±  0.51cd | 7.24±  1.77d | 10.24±  2.8bc |
| 45 | 1-Nonanol | Citrus odor | 143-08-8 | Alcohol | 1.41145 | 25.75±  0.96a | 24.75±  0.66a | 21.88±  1.50b | 15.37±  1.82c | 11.55±  1.83d | 12.08±  0.99d |
| 46 | 3,6-Octadienal,  3,7-dimethyl- | nd | 55722-59-3 | Aldehyde | 1.28898 | 71.35±  1.15a | 25.97±  0.27b | 72.97±  3.01a | 4.26±  0.28c | 2.85±  0.38c | 3.30±  0.31c |
| 47 | Methyl salicylate | Minty, wintergreen-like | 119-36-8 | Ester | 0.254053 | 327.46±  1.50d | 406.71±  2.10b | 302.75±  1.10f | 455.22±  5.02a | 311.75±  2.51e | 337.12±  1.70c |
| 48 | .alpha.-Terpineol | Pleasant, floral | 98-55-5 | Terpene Alcohol | 0.557518 | 12.44±  1.06a | 10.34±  1.5ab | 9.78±  0.57b | 11.18±  1.35ab | 9.48±  0.96b | 11.17±  1.5ab |
| 49 | 1,3-Cyclohexadiene-1-carboxaldehyde,  2,6,6-trimethyl- | Herbal type odor | 116-26-7 | Aldehyde | 1.29256 | 17.24±  0.33c | 21.38±  0.54b | 27.75±  0.56a | 9.32±  2.52d | 9.94±  1.32d | 10.04±  1.00d |
| 50 | Undecane, 4,6-dimethyl- | nd | 17312-82-2 | Hydrocarbon | 1.32197 | 7.15±  1.69a | 4.85±  0.78b | 4.70±  0.52b | 1.80±  0.47c | 1.63±  0.33c | 1.97±  0.38c |
| 51 | Coumarin, 3,4-dihydro-4,5,7-trimethyl- | nd | 1000126-60-5 | Lactone | 0.388991 | 0.55±  0.24a | 0.72±  0.16a | 0.80±  0.26a | 0.53±  0.07a | 0.81±  0.32a | 0.65±  0.20a |
| 52 | Decanal | Sweet, aldehydic, fresh, orange, waxy and floral | 112-31-2 | Aldehyde | 1.27302 | 5.26±  0.35a | 3.22±  1.02b | 1.91±  0.72c | 1.05±  0.22c | 0.96±  0.11c | 1.19±  0.41c |
| 53 | 1-Cyclohexene-1-carboxaldehyde, 2,6,6-trimethyl- | nd | 432-25-7 | Aldehyde | 1.13622 | 11.48±  0.58c | 16.15±  0.91b | 17.92±  0.51a | 9.15±  1.20d | 8.14±  1.01d | 9.78±  1.06d |
| 54 | Furan, 3-phenyl- | Caramel | 13679-41-9 | Furan | 0.233396 | 1.93±  0.80c | 3.81±  0.20a | 2.56±  0.07bc | 3.62±  0.65a | 3.23±  0.66ab | 3.52±  0.23a |
| 55 | 6-Octen-1-ol,  7-methyl-3-methylene- | nd | 13066-51-8 | Alcohol | 0.512586 | 10.95±  0.50ab | 11.2±  0.07ab | 11.69±  0.27a | 12.31±  1.86a | 9.69±  0.93b | 11.7±  0.52a |
| 56 | 2,6-Octadien-1-ol,  3,7-dimethyl-, (Z)- | Floral | 106-25-2 | Terpene Alcohol | 0.82759 | 112.62±  0.60c | 115.75±  0.60b | 116.16±  1.00b | 91.78±  0.70d | 85.75±  0.55e | 117.75±  1.20a |
| 57 | 3,6-Octadien-1-ol,  3,7-dimethyl-, (Z)- | Sweet-rosy | 5944-20-7 | Terpene Alcohol | 1.00349 | 3.66±  1.62a | 4.26±  0.63a | 4.19±  0.32a | 3.27±  0.52a | 2.72±  0.24a | 3.37±  0.64a |
| 58 | n-Valeric acid cis-3-hexenyl ester | Green fruity apple pear | 35852-46-1 | Acid | 1.39713 | 20.74±  0.49a | 20.46±  1.18a | 18.10±  11.7a | 12.71±  1.80ab | 8.05±  1.07b | 6.29±  0.88b |
| 59 | Neral | Vibrant lemony aroma | 106-26-3 | Aldehyde |  | 13.77±  0.56d | 12.48±  1.53d | 12.25±  0.26d | 38.79±  1.16b | 32.08±  1.81c | 42.12±  1.13a |
| 60 | 2,2-Dimethylpropanoic anhydride | nd | 1538-75-6 | Acid | 1.43917 | 11.6±  1.07b | 15.76±  1.09a | 11.46±  0.58b | 5.43±  0.81c | 2.46±  0.86d | 3.34±  0.47d |
| 61 | 1-Cyclohexene-1-acetaldehyde,  2,6,6-trimethyl- | Oily; woody; cooling; camphoraceous; fruity | 472-66-2 | Aldehyde | 1.10996 | 2.58±  0.39b | 4.46±  0.53a | 4.37±  0.54a | 2.33±  0.22b | 2.30±  0.31b | 2.46±  0.45b |
| 62 | Geraniol | Rose-like, sweet, honey-like | 106-24-1 | Terpene Alcohol | 0.654784 | 2221.5±  6.90d | 2208.2±  0.20d | 1962.80±  2.80e | 3753.30±  10.90a | 2956.0±  19.20c | 3565.30±  4.00b |
| 63 | 2-Decenal, (E)- | Sweet, green odor | 3913-81-3 | Aldehyde | 0.715554 | 178.90±  1.24d | 178.0±  3.68d | 158.5±  3.14e | 314.32±  2.95a | 247.75±  1.09c | 300.7±  1.15b |
| 64 | Benzeneacetaldehyde,  .alpha.-ethylidene- | nd | 4411-89-6 | Aldehyde | 0.174326 | 10.46±  0.57b | 11.72±  0.59b | 10.44±  0.52b | 12.26±  1.67b | 12.28±  2.13b | 15.52±  0.38a |
| 65 | 2,6-Octadienal,  3,7-dimethyl-, (E)- | Citrus type odor | 141-27-5 | Aldehyde | 1.20064 | 43.2±  0.88e | 424.27±  1.6d | 28.71±  1.23f | 2517.10±  6.08a | 2023.80±  4.08b | 1890.5±  9.30c |
| 66 | Dodecane, 4,6-dimethyl- | Mild fresh green | 61141-72-8 | Hydrocarbon | 1.21686 | 115.13±  2.80c | 107.46±  2.40b | 128.72±  0.50a | 71.60±  2.60d | 39.53±  1.64f | 55.65±  0.98e |
| 67 | Sulfurous acid,  dodecyl 2-ethylhexyl ester | nd | 1000309-19-5 | Acid | 1.25427 | 16.26±  1.40ab | 15.38±  1.03b | 17.62±  0.76a | 10.78±  1.26c | 5.26±  0.61e | 8.13±  0.90d |
| 68 | 3-Methyl-4-isopropylphenol | nd | 2/2/3228 | Phenol | 0.913194 | 32.79±  2.52a | 3.78±  0.59b | 2.33±  0.18bc | 1.24±  0.23c | 1.11±  0.15c | 1.8±  0.49bc |
| 69 | 3-Buten-2-one,  4-(2,6,6-trimethyl-1-cyclohexen-1-yl)- | Strong aroma of violet, raspberry | 14901-07-6 | Ketone | 1.00555 | 0.95±  0.41c | 1.55±  0.84bc | 1.10±  0.47c | 2.56±  0.23ab | 2.81±  0.66ab | 3.55±  1.08a |
| 70 | 1-Oxaspiro[4.5]dec-  6-ene, 2,6,10,10-tetramethyl- | Herbal, greeen tea, moist and recently cut tobacco leaves, with metallic, woody, floral and spicy backnotes | 36431-72-8 | Terpene | 1.13622 | 1.00±  0.47b | 0.74±  0.18b | 0.74±  0.40b | 3.34±  0.89a | 2.97±  0.84a | 2.68±  0.85a |
| 71 | 2-Isopropyl-5-methyl-  1-heptanol | nd | 91337-07-4 | others | 1.43869 | 1.28±  0.62a | 1.22±  0.43a | 1.28±  0.68a | 0.54±  0.19ab | 0.36±  0.11b | 0.49±  0.10ab |
| 72 | 2-Bromo dodecane | nd | 13187-99-0 | others | 1.1582 | 10.42±  1.01b | 10.28±  0.62b | 12.65±  1.50a | 6.12±  0.96c | 3.64±  0.81d | 5.85±  0.75c |
| 73 | Pentadecane | Waxy Odor | 629-62-9 | Hydrocarbon | 1.36843 | 39.41±  2.58a | 38.68±  1.90a | 40.28±  1.61a | 22.16±  2.00b | 12.19±  1.16d | 18.18±  2.29c |
| 74 | Nonane,  3-methyl-5-propyl- | nd | 31081-18-2 | Hydrocarbon | 1.42157 | 8.82±  1.61a | 7.62±  1.88a | 7.48±  0.33a | 4.25±  1.14b | 2.37±  0.76b | 3.67±  0.54b |
| 75 | Benzeneacetaldehyde,  .alpha.(2methyl-propylidene)- | Ethereal odor | 26643-91-4 | Aldehyde | 0.056153 | 2.17±  0.96a | 3.29±  1.74a | 2.23±  0.83a | 2.65±  0.55a | 2.82±  0.56a | 3.31±  1.45a |
| 76 | Propanoic acid,  2-methyl-,  3-hydroxy-  2,2,4-trimethylpentyl ester | Cheesy and rancid | 77-68-9 | Acid | 1.24217 | 3.66±  0.35a | 3.77±  0.69a | 3.44±  1.91ab | 1.44±  0.19c | 1.48±  0.15c | 1.96±  0.73bc |
| 77 | 2-Buten-1-one,  1-(2,6,6-trimethyl-  1,3-cyclohexadien-1-yl)-, (E)- | Rose, fruity | 23726-93-4 | Ketone | 0.507224 | 4.00±  1.82c | 8.48±  0.58a | 6.67±  0.60b | 2.70±  0.32c | 3.80±  1.04c | 3.79±  0.72c |
| 78 | Hexanoic acid,  3-hexenyl ester, (Z)- | Fruity | 31501-11-8 | Acid | 1.17897 | 32.78±  0.68a | 33.46±  2.08a | 23.09±  2.06b | 18.45±  1.58c | 14.01±  1.12d | 9.63±  0.74e |
| 79 | 2-Cyclopenten-1-one,  3-methyl-2-(2-pentenyl)-, (Z)- | Fruity and minty effect. | 488-10-8 | other | 0.568651 | 3.20±  0.74b | 4.69±  0.65a | 1.73±  0.59cd | 2.35±  0.29bcd | 1.48±  0.67d | 2.81±  0.97bc |
| 80 | Hexanoic acid,  2-hexenyl ester, (E)- | Green type odor | 53398-86-0 | Acid | 1.09686 | 9.80±  1.05b | 12.14±  1.99a | 6.97±  1.03c | 3.32±  0.71d | 2.69±  0.15d | 1.99±  0.60d |
| 81 | Tetradecane | Mild waxy. | 629-59-4 | Hydrocarbon | 1.36881 | 9.51±  0.78a | 5.89±  0.67b | 6.44±  0.38b | 3.67±  1.04c | 2.56±  1.08c | 2.93±  0.24c |
| 82 | .alpha.-Ionone | Floral, violet-like, powdery, berry-like | 127-41-3 | Ketone | 1.17167 | 5.37±  0.57b | 7.82±  0.31a | 5.30±  1.12b | 3.27±  0.44c | 2.59±  0.21c | 3.91±  1.08c |
| 83 | Butylphosphonic acid, di(2-phenylethyl) ester | Floral | 1000315-10-4 | Acid | 0.257659 | 1.52±  0.49b | 2.69±  0.22a | 1.34±  0.77b | 2.06±  0.43ab | 1.45±  0.18b | 1.74±  0.51b |
| 84 | 5,9-Undecadien-2-one, 6,10-dimethyl- | Fresh rose leafy floral green | 689-67-8 | Ketone | 0.836279 | 1.98±  0.69b | 4.10±  1.8a | 1.68±  0.72b | 1.44±  0.26b | 1.06±  0.26b | 1.54±  0.61b |
| 85 | trans-.beta.-Ionone | Cedar wood | 79-77-6 | Ketone | 1.12807 | 59.26±  2.30b | 96.78±  2.34a | 57.47±  1.55b | 36.02±  0.98c | 27.41±  2.7d | 34.01±  4.47c |
| 86 | 2-Fluorobenzoic acid,  4-nitrophenyl ester | nd | 1000307-69-1 | other | 1.16605 | 20.94±  0.9b | 32.63±  0.67a | 19.39±  1.47b | 11.65±  1.72c | 7.18±  1.62d | 13.25±  2.08c |
| 87 | Tridecanol,  2-ethyl-2-methyl- | Pleasant odor | 1010115-66-1 | Alcohol | 1.49685 | 6.33±  0.57a | 7.61±  0.91a | 6.70±  3.29a | 3.61±  0.9b | 2.02±  0.6b | 3.12±  0.25b |
| 88 | 2,4-Di-tert-butylphenol | Phenolic' and 'herbal' odors in Fu-brick tea, | 96-76-4 | Phenol | 0.854572 | 515.90±  1.13a | 15.56±  2.42b | 11.59±  5.00bc | 5.96±  1.01d | 5.27±  0.97d | 7.79±  2.29cd |
| 89 | Eicosyl octyl ether | nd | 1000406-38-8 | Hydrocarbon | 1.50392 | 3.58±  1.42ab | 4.08±  0.51a | 3.53±  1.8ab | 1.79±  0.43bc | 0.99±  0.32c | 1.44±  0.18c |
| 90 | Heptadecane, 2,6,10,15-tetramethyl- | nd | 54833-48-6 | Hydrocarbon | 1.5158 | 4.71±  1.55a | 5.03±  0.56a | 4.29±  1.89a | 2.36±  0.50b | 1.25±  0.40b | 2.00±  0.05b |
| 91 | Hexadecane,  2,6,11,15-tetramethyl- | nd | 504-44-9 | Hydrocarbon | 1.51624 | 32.73±  4.02a | 36.63±  3.91a | 31.11±  2.02a | 16.08±  4.07b | 9.02±  3.39c | 14.09±  0.80bc |
| 92 | 1,6,10-Dodecatrien-3-ol, 3,7,11-trimethyl-, (E)- | Waxy Odor | 40716-66-3 | Terpene Alcohol | 0.332235 | 7.50±  2.45ab | 9.54±  2.35a | 4.73±  1.63b | 7.23±  1.06ab | 6.49±  2.92ab | 5.74±  1.61ab |
| 93 | 2,2,4-Trimethyl-  1,3-pentanediol diisobutyrate | Musty odor | 6846-50-0 | Ester | 1.38095 | 6.28±  2.93ab | 7.53±  3.05a | 6.07±  2.5abc | 2.67±  0.75bc | 2.21±  0.30c | 2.76±  0.96bc |
| 94 | Cedrol | Cedar, sandalwood note | 77-53-2 | Terpene Alcohol | 1.38397 | 2.49±  1.52a | 2.25±  0.73ab | 1.74±  0.70abc | 0.94±  0.19bc | 0.66±  0.05c | 0.92±  0.29bc |
| 95 | Dodecanoic acid, ethyl ester | Sweet, waxy, soapy and rummy with a creamy, floral nuance | 106-33-2 | Acid | 0.140226 | 2.44±  1.99a | 1.84±  0.39a | 1.47±  0.81a | 1.89±  0.38a | 1.32±  0.94a | 3.43±  1.79a |
| 96 | l-Alanine,  N-(4-butylbenzoyl)-, isobutyl ester | nd | 1000314-15-2 | Acid | 0.896435 | 2.22±  0.82b | 2.75±  0.22a | 1.05±  0.26c | 0.67±  0.20c | 0.48±  0.12c | 0.63±  0.22c |
| 97 | Heneicosane | nd | 629-94-7 | Hydrocarbon | 1.48448 | 12.38±  3.13a | 13.83±  4.12a | 10.56±  4.65a | 5.11±  1.30b | 3.01±  0.97b | 4.68±  0.46b |
| 98 | 1,2-  Benzenedicarboxylic acid, bis(2-methylpropyl) ester | nd | 84-69-5 | Acid | 1.33603 | 25.54±  2.05a | 24.88±  1.11a | 13.39±  8.48b | 6.38±  1.17c | 4.35±  0.93c | 6.16±  0.62c |

**nd** stands for not defined (unknown). Different lower-case letters indicate significant differences among samples of each cultivar The data was shown as the mean values ± standard deviation.*****

**Table S4**. VOCs VIP >1 detected by GC-MS

| # | VOCs | VIP values | Category |
| --- | --- | --- | --- |
|  | Hexadecane, 2,6,11,15-tetramethyl- | 1.52 | Hydrocarbon |
|  | Heptadecane, 2,6,10,15-tetramethyl- | 1.52 | Hydrocarbon |
|  | Eicosyl octyl ether | 1.50 | Hydrocarbon |
|  | Tridecanol, 2-ethyl-2-methyl- | 1.50 | Alcohol |
|  | Hexadecane | 1.49 | Hydrocarbon |
|  | Heneicosane | 1.48 | Hydrocarbon |
|  | 2,2-Dimethylpropanoic anhydride | 1.44 | acid |
|  | 2-Isopropyl-5-methyl-1-heptanol | 1.44 | other |
|  | Nonane, 3-methyl-5-propyl- | 1.42 | Hydrocarbon |
|  | 1-Nonanol | 1.41 | Alcohol |
|  | Undecane, 5,7-dimethyl- | 1.41 | Hydrocarbon |
|  | n-Valeric acid cis-3-hexenyl ester | 1.40 | acid |
|  | Cedrol | 1.38 | terpene alcohol |
|  | 2,2,4-Trimethyl-1,3-pentanediol diisobutyrate | 1.38 | ester |
|  | Nonanal | 1.37 | aldehyde |
|  | Tetradecane | 1.37 | Hydrocarbon |
|  | Pentadecane | 1.37 | Hydrocarbon |
|  | Formic acid, octyl ester | 1.34 | acid |
|  | 1,2-Benzenedicarboxylic acid, bis(2-methylpropyl) ester | 1.34 | acid |
|  | Undecane, 4,6-dimethyl- | 1.32 | Hydrocarbon |
|  | Linalool | 1.32 | terpene alcohol |
|  | Hexanal | 1.31 | aldehyde |
|  | 1,3-Cyclohexadiene-1-carboxaldehyde, 2,6,6-trimethyl- | 1.29 | aldehyde |
|  | 3,6-Octadienal, 3,7-dimethyl- | 1.29 | aldehyde |
|  | Decanal | 1.27 | aldehyde |
|  | (3R,6S)-2,2,6-Trimethyl-6-vinyltetrahydro-2H-pyran-3-ol | 1.26 | terpene alcohol |
|  | Acetophenone | 1.26 | ketone |
|  | 1-Heptanol | 1.26 | alcohol |
|  | Sulfurous acid, dodecyl 2-ethylhexyl ester | 1.25 | acid |
|  | Heptanal | 1.25 | aldehyde |
|  | Propanoic acid, 2-methyl-, 3-hydroxy-2,2,4-trimethylpentyl ester | 1.24 | acid |
|  | Dodecane, 4,6-dimethyl- | 1.22 | Hydrocarbon |
|  | 2,6-Octadienal, 3,7-dimethyl-, (E)- | 1.20 | ketone |
|  | Hexanoic acid, 3-hexenyl ester, (Z)- | 1.18 | acid |
|  | .alpha.-Ionone | 1.17 | ketone |
|  | Octanal | 1.17 | aldehyde |
|  | 2-Fluorobenzoic acid, 4-nitrophenyl ester | 1.17 | other |
|  | 2-Bromo dodecane | 1.16 | other |
|  | 1-Cyclohexene-1-carboxaldehyde, 2,6,6-trimethyl- | 1.14 | aldehyde |
|  | Neral | 1.14 | aldehyde |
|  | 1-Oxaspiro[4.5]dec-6-ene, 2,6,10,10-tetramethyl- | 1.14 | terpene |
|  | 2-Furanmethanol, 5-ethenyltetrahydro-.alpha.,.alpha.,5-trimethyl-, cis- | 1.14 | furan |
|  | trans-.beta.-Ionone | 1.13 | ketone |
|  | 1-Cyclohexene-1-acetaldehyde, 2,6,6-trimethyl- | 1.11 | aldehyde |
|  | Hexanoic acid, 2-hexenyl ester, (E)- | 1.10 | acid |
|  | Nonane, 4,5-dimethyl- | 1.01 | Hydrocarbon |
|  | 2,4-Heptadienal, (E,E)- | 1.01 | aldehyde |
|  | 3-Buten-2-one, 4-(2,6,6-trimethyl-1-cyclohexen-1-yl)- | 1.01 | ketone |
|  | 3,6-Octadien-1-ol, 3,7-dimethyl-, (Z)- | 1.00 | terpene alcohol |
|  | 1,6-Heptadiene, 3-methyl- | 1.00 | Hydrocarbon |

Note: ‘other’ the volatile compounds were not from the alcohols, terpene alcohols, aldehydes, ketones, hydrocarbons, acids, furans and ester compounds.

Table S5. VOCs with OAV> 1

| # | VOCs | | OAVs | |  | |  | |  | |  | |  | |
| --- | --- | --- | --- | --- | --- | --- | --- | --- | --- | --- | --- | --- | --- | --- |
|  |  |  | B2W3 | | B2W6 | | B2W9 | | B2J3 | | B2J6 | | B2J9 | |
| 1 | Hexanal | | 15.16 | | 14.90 | | 16.97 | | 8.69 | | 6.06 | | 9.64 | |
| 2 | 2-Propenal | | 530.39 | | 418.92 | | 648.88 | | 399.3 | | 354.97 | | 347.36 | |
| 3 | Heptanal | | 4.45 | | 2.95 | | 3.41 | | 1.99 | | 1.95 | | 2.10 | |
| 4 | 5-Hepten-2-one, 6-methyl- | | 12.16 | | 11.77 | | 14.85 | | 16.85 | | 16.00 | | 19.11 | |
| 5 | .beta.-Myrcene | | 50.43 | | 47.33 | | 36.48 | | 60.7 | | 44.39 | | 57.23 | |
| 6 | Octanal | | 2.23 | | 1.27 | | 1.35 | | 0.79 | | 0.67 | | 1.08 | |
| 7 | Benzeneacetaldehyde | | 190.52 | | 172.17 | | 219.9 | | 214.55 | | 217.67 | | 250.39 | |
| 8 | 2-Butenal | | 21.86 | | 15.18 | | 24.88 | | 14.46 | | 10.17 | | 17.75 | |
| 9 | 2Furanmethanol,5ethenyltetrahydro-.alpha.,.alpha.,5-trimethyl-, cis- | | 1.75 | | 1.72 | | 2.12 | | 1.27 | | 1.11 | | 1.13 | |
| 10 | Linalool | | 12333.72 | | 11785.29 | | 13510.25 | | 8065.03 | | 5838.68 | | 5429.84 | |
| 11 | Nonanal | | 30.73 | | 22.34 | | 22.22 | | 12.28 | | 9.21 | | 11.38 | |
| 12 | (3R,6S)-2,2,6-Trimethyl-6-vinyltetrahydro-2H-pyran-3-ol | | 25.92 | | 22.14 | | 23.55 | | 16.17 | | 12.92 | | 18.29 | |
| 13 | 3,6-Octadienal, 3,7-dimethyl- | | 837.46 | | 301.07 | | 849.16 | | 50.12 | | 33.52 | | 36.71 | |
| 14 | Methyl salicylate | | 9636.47 | | 11945.52 | | 8908.7 | | 13384.4 | | 9165.33 | | 9912.39 | |
| 15 | .alpha.-Terpineol | | 141.2 | | 120.22 | | 114.2 | | 130.05 | | 110.24 | | 132.59 | |
| 16 | 1-Cyclohexene-1-carboxaldehyde, 2,6,6-trimethyl- | | 2.23 | | 3.20 | | 3.49 | | 1.83 | | 1.63 | | 1.82 | |
| 17 | Geraniol | | 2020.56 | | 2008.97 | | 1784.22 | | 3392.2 | | 2686.62 | | 3242.63 | |
| 18 | 2,6-Octadienal, 3,7-dimethyl-, (E)- | | 1.37 | | 13.26 | | 0.89 | | 78.66 | | 63.31 | | 59.25 | |
| 19 | .alpha.-Ionone | | 13.39 | | 19.42 | | 13.5 | | 8.18 | | 6.48 | | 9.78 | |
| 20 | trans-.beta.-Ionone | | 657.13 | | 1073.26 | | 640.09 | | 406.67 | | 304.55 | | 378.45 | |
| 21 | Cedrol | | 4.97 | | 4.50 | | 3.47 | | 1.88 | | 1.31 | | 1.85 | |
|  | |  | |  | |  | |  | |  | |  | |  |

**Table S6.** VOCs detected by GC-IMS of black tea cultivars processed with varying fermentation times followed by drying at 95℃

| Count # | Compound | CAS# | Formula | MW | RI | Rt [sec] | Dt [a.u.] | OT | Contents ug/g | | | | | | Identification |
| --- | --- | --- | --- | --- | --- | --- | --- | --- | --- | --- | --- | --- | --- | --- | --- |
|  |  |  |  |  |  |  |  |  | B2W3 | B2W6 | B2W9 | B2J3 | B2J6 | B2J9 |  |
| 1 | Borneol | 507-70-0 | C10H18O | 154.3 | 1734.5 | 1569.649 | 1.21498 | 0.14 | 1.02 | 1.07 | 1.06 | 1.00 | 1.15 | 0.97 | RI, Dt |
| 2 | (phenylacetaldehyde | 122-78-1 | C8H8O | 120.2 | 1646.1 | 1293.594 | 1.25232 | 0.0003 | 0.89 | 0.79 | 0.63 | 1.24 | 1.55 | 1.02 | RI, Dt |
| 3 | 1-ethyl-1H-pyrrole-2-carboxaldehyde | 2167-14-8 | C7H9NO | 123.2 | 1606.1 | 1185.36 | 1.17457 | nd | 2.02 | 2.12 | 1.63 | 0.75 | 0.88 | 0.72 | RI, Dt |
| 4 | Menthyl acetate | 89-48-5 | C12H22O2 | 198.3 | 1576.4 | 1110.761 | 1.22832 | nd | 2.37 | 1.85 | 1.05 | 1.82 | 1.62 | 0.93 | RI, Dt |
| 5 | Propanoic acid | 79-09-4 | C3H6O2 | 74.1 | 1574.3 | 1105.718 | 1.1039 | 0.1 | 1.30 | 1.35 | 1.16 | 1.62 | 1.76 | 1.83 | RI, Dt |
| 6 | Benzaldehyde-M | 100-52-7 | C7H6O | 106.1 | 1518 | 977.499 | 1.15664 | 0.024 | 2.23 | 1.94 | 2.06 | 2.53 | 2.62 | 2.31 | RI, Dt |
| 7 | Benzaldehyde-D | 100-52-7 | C7H6O | 106.1 | 1516.9 | 975.179 | 1.48007 | 0.024 | 0.35 | 0.26 | 0.29 | 0.65 | 0.71 | 0.57 | RI, Dt |
| 8 | Acetic acid-M | 64-19-7 | C2H4O2 | 60.1 | 1483.8 | 906.978 | 1.05292 | 22 | 13.8 | 12.59 | 12.64 | 10.76 | 11.11 | 10.16 | RI, Dt |
| 9 | Acetic acid-D | 64-19-7 | C2H4O2 | 60.1 | 1482.8 | 905.122 | 1.15218 | 22 | 9.97 | 11.4 | 11.54 | 9.01 | 10.24 | 9.58 | RI, Dt |
| 10 | 2,3-Diethyl-5-methylpyrazine | 18138-04-0 | C9H14N2 | 150.2 | 1514.9 | 970.918 | 1.29533 | 0.000031 | 0.40 | 0.39 | 0.39 | 0.46 | 0.48 | 0.43 | RI, Dt |
| 11 | Dihydromyrcenol | 53219-21-9 | C10H20O | 156.3 | 1478.7 | 897.057 | 1.22687 | nd | 0.18 | 0.22 | 0.20 | 0.31 | 0.31 | 0.34 | RI, Dt |
| 12 | furan linalool oxide-M | 60047-17-8 | C10H18O2 | 170.3 | 1470.3 | 880.644 | 1.2698 | nd | 0.98 | 0.76 | 0.57 | 0.74 | 0.78 | 0.50 | RI, Dt |
| 13 | furan linalool oxide-D | 60047-17-8 | C10H18O2 | 170.3 | 1471.3 | 882.509 | 1.81742 | nd | 0.19 | 0.13 | 0.08 | 0.14 | 0.16 | 0.07 | RI, Dt |
| 14 | Tetrahydrolinalool | 78-69-3 | C10H22O | 158.3 | 1445 | 833.268 | 1.264 | nd | 0.93 | 0.70 | 0.51 | 0.62 | 0.65 | 0.43 | RI, Dt |
| 15 | ( Z)-3-hexen-1-ol-M | 928-96-1 | C6H12O | 100.2 | 1404.8 | 763.137 | 1.22803 | 0.0039 | 3.43 | 3.07 | 2.60 | 2.71 | 2.61 | 2.33 | RI, Dt |
| 16 | ( Z)-3-hexen-1-ol-D | 928-96-1 | C6H12O | 100.2 | 1403.4 | 760.778 | 1.51305 | 0.0039 | 3.47 | 2.87 | 1.91 | 3.46 | 2.92 | 1.72 | RI, Dt |
| 17 | (E)-2-Hexen-1-ol-M | 928-95-0 | C6H12O | 100.2 | 1427.2 | 801.446 | 1.3545 | 0.1 | 0.29 | 0.26 | 0.13 | 0.28 | 0.27 | 0.15 | RI, Dt |
| 18 | (E)-2-Hexen-1-ol-D | 928-95-0 | C6H12O | 100.2 | 1426.2 | 799.595 | 1.8171 | 0.1 | 0.18 | 0.17 | 0.09 | 0.25 | 0.21 | 0.10 | RI, Dt |
| 19 | 1-hexanol-M | 111-27-3 | C6H14O | 102.2 | 1378.1 | 719.817 | 1.32439 | 0.0056 | 2.88 | 2.52 | 1.96 | 2.66 | 2.59 | 1.73 | RI, Dt |
| 20 | 1-hexanol-D | 111-27-3 | C6H14O | 102.2 | 1377.6 | 719.071 | 1.63661 | 0.0056 | 0.64 | 0.54 | 0.31 | 0.70 | 0.60 | 0.32 | RI, Dt |
| 21 | 2-methyl-2-hepten-6-one | 110-93-0 | C8H14O | 126.2 | 1359.2 | 690.739 | 1.18079 | 0.05 | 0.95 | 0.83 | 0.71 | 1.53 | 2.19 | 1.68 | RI, Dt |
| 22 | 1-pentanol-4-methyl-M | 626-89-1 | C6H14O | 102.2 | 1347.8 | 673.59 | 1.63793 | 0.82 | 1.61 | 1.14 | 0.84 | 1.55 | 1.13 | 0.63 | RI, Dt |
| 23 | 1-pentanol-4-methyl-D | 626-89-1 | C6H14O | 102.2 | 1347.2 | 672.845 | 1.32702 | 0.82 | 1.41 | 1.46 | 1.25 | 1.09 | 1.15 | 1.21 | RI, Dt |
| 24 | (Z)-2-Penten-1-ol | 1576-95-0 | C5H10O | 86.1 | 1346.7 | 672.099 | 1.45349 | 0.72 | 0.57 | 0.42 | 0.39 | 0.58 | 0.55 | 0.39 | RI, Dt |
| 25 | 1-Hydroxy-2-propanone-D | 116-09-6 | C3H6O2 | 74.1 | 1320.4 | 634.447 | 1.23349 | 10 | 1.50 | 1.79 | 1.45 | 1.42 | 1.63 | 1.51 | RI, Dt |
| 26 | 1-Hydroxy-2-propanone-M | 116-09-6 | C3H6O2 | 74.1 | 1320.5 | 634.642 | 1.03937 | 10 | 1.96 | 1.84 | 1.73 | 1.34 | 1.22 | 1.10 | RI, Dt |
| 27 | (Z)-3-hexenyl propanoate | 33467-74-2 | C9H16O2 | 156.2 | 1403.9 | 761.688 | 1.3719 | nd | 0.45 | 0.48 | 0.36 | 0.30 | 0.34 | 0.36 | RI, Dt |
| 28 | (E)-2-Heptenal | 18829-55-5 | C7H12O | 112.2 | 1343.9 | 667.987 | 1.24769 | 0.013 | 0.20 | 0.53 | 0.35 | 0.16 | 0.26 | 0.92 | RI, Dt |
| 29 | 2-Methyl-2-cyclopenten-1-one | 1120-73-6 | C6H8O | 96.1 | 1378.7 | 720.731 | 1.42035 | nd | 0.31 | 0.33 | 0.20 | 0.25 | 0.34 | 0.27 | RI, Dt |
| 30 | 2-Methyl-1-pentanol | 105-30-6 | C6H14O | 102.2 | 1304 | 612.058 | 1.28419 | 0.83 | 0.29 | 0.34 | 0.27 | 0.22 | 0.27 | 0.29 | RI, Dt |
| 31 | 2-Butanone, 3-hydroxy-D | 513-86-0 | C4H8O2 | 88.1 | 1303.5 | 611.436 | 1.3347 | 0.014 | 0.32 | 0.30 | 0.27 | 0.27 | 0.26 | 0.24 | RI, Dt |
| 32 | 2-Butanone, 3-hydroxy-M | 513-86-0 | C4H8O2 | 88.1 | 1305.1 | 613.615 | 1.05688 | 0.014 | 0.88 | 0.98 | 0.68 | 0.67 | 0.67 | 0.66 | RI, Dt |
| 33 | 1-Pentanol-M | 71-41-0 | C5H12O | 88.1 | 1266.5 | 546.367 | 1.25392 | 0.12 | 3.42 | 3.22 | 2.51 | 2.53 | 2.67 | 2.65 | RI, Dt |
| 34 | 2-Methylpyridine | 109-06-8 | C6H7N | 93.1 | 1265.7 | 544.975 | 1.35004 | 0.01 | 2.10 | 2.08 | 1.51 | 1.60 | 1.88 | 2.20 | RI, Dt |
| 35 | ethyl 2-oxopropanoate | 617-35-6 | C5H8O3 | 116.1 | 1270.2 | 553.05 | 1.43615 | 5 | 0.78 | 0.48 | 0.93 | 0.44 | 0.58 | 0.47 | RI, Dt |
| 36 | 1-Pentanol-D | 71-41-0 | C5H12O | 88.1 | 1266.7 | 546.647 | 1.51088 | 0.12 | 1.45 | 1.16 | 1.05 | 1.51 | 1.36 | 1.24 | RI, Dt |
| 37 | ( E)-2-hexen-1-al-M | 6728-26-3 | C6H10O | 98.1 | 1236.6 | 495.118 | 1.18669 | 0.01 | 1.65 | 1.35 | 1.39 | 1.51 | 1.37 | 1.22 | RI, Dt |
| 38 | Isoamyl butyrate | 2050-01-3 | C9H18O2 | 158.2 | 1233.6 | 490.239 | 1.38231 | 0.087 | 2.82 | 2.85 | 2.35 | 1.60 | 1.99 | 2.26 | RI, Dt |
| 39 | ( E)-2-hexen-1-al-D | 6728-26-3 | C6H10O | 98.1 | 1232.1 | 487.8 | 1.52187 | 0.01 | 5.75 | 5.03 | 3.64 | 7.58 | 8.65 | 6.67 | RI, Dt |
| 40 | 1,2-dimethylbenzene | 95-47-6 | C8H10 | 106.2 | 1218.3 | 466.096 | 1.07098 | 0.45023 | 3.19 | 3.01 | 3.28 | 2.28 | 2.56 | 2.59 | RI, Dt |
| 41 | Pyridine | 110-86-1 | C_5_H_5_N | 79.1 | 1194.9 | 431.468 | 1.24351 | 0.00003 | 27.03 | 28.56 | 25.39 | 20.21 | 26.18 | 26.85 | RI, Dt |
| 42 | Heptaldehyde | 111-71-7 | C_7_H_14_O | 114.2 | 1220 | 468.675 | 1.3423 | 0.00025 | 0.99 | 0.75 | 0.52 | 0.98 | 0.86 | 0.58 | RI, Dt |
| 43 | (E)-2-Pentenal-M | 1576-87-0 | C_5_H_8_O | 84.1 | 1150.6 | 369.835 | 1.10904 | 0.31 | 1.02 | 0.91 | 0.85 | 0.79 | 0.80 | 0.77 | RI, Dt |
| 44 | 1- butanol-M | 71-36-3 | C_4_H_10_O | 74.1 | 1159.1 | 380.919 | 1.18107 | 0.27 | 1.38 | 1.24 | 1.19 | 1.01 | 1.08 | 1.00 | RI, Dt |
| 45 | 2-Butylfuran | 4466-24-4 | C_8_H_12_O | 124.2 | 1140.6 | 357.12 | 1.17733 | 0.005 | 1.00 | 0.89 | 0.78 | 0.54 | 0.56 | 0.47 | RI, Dt |
| 46 | 1- butanol-D | 71-36-3 | C_4_H_10_O | 74.1 | 1157.1 | 378.311 | 1.38408 | 0.27 | 1.10 | 0.91 | 0.81 | 0.87 | 0.96 | 0.88 | RI, Dt |
| 47 | 3-penten-2-one, 4-methyl | 141-79-7 | C_6_H_10_O | 98.1 | 1148.8 | 367.553 | 1.45144 | 0.07 | 2.80 | 2.31 | 1.42 | 1.02 | 1.06 | 0.75 | RI, Dt |
| 48 | 1-Propanol, 2-methyl-M | 78-83-1 | C_4_H_10_O | 74.1 | 1107.9 | 318.65 | 1.17172 | 0.36 | 0.66 | 0.54 | 0.48 | 0.36 | 0.38 | 0.29 | RI, Dt |
| 49 | 1-hexanal-M | 66-25-1 | C_6_H_12_O | 100.2 | 1097.3 | 306.995 | 1.26683 | 0.00032 | 1.89 | 1.62 | 1.52 | 1.03 | 1.13 | 0.89 | RI, Dt |
| 50 | 1-hexanal-D | 66-25-1 | C_6_H_12_O | 100.2 | 1099.8 | 309.658 | 1.5684 | 0.00032 | 1.96 | 1.43 | 1.28 | 2.18 | 2.13 | 2.40 | RI, Dt |
| 51 | 1-Propanol, 2-methyl-D | 78-83-1 | C_4_H_10_O | 74.1 | 1107.2 | 317.872 | 1.38824 | 0.36 | 1.26 | 1.07 | 0.79 | 0.76 | 0.74 | 0.60 | RI, Dt |
| 52 | 2-Hexanone | 591-78-6 | C_6_H_12_O | 100.2 | 1103.4 | 313.66 | 1.48972 | 0.04 | 0.31 | 0.23 | 0.17 | 0.34 | 0.31 | 0.29 | RI, Dt |
| 53 | Dibutylamine | 111-92-2 | C_8_H_19_N | 129.2 | 1106.1 | 316.623 | 1.73133 | 0.42 | 0.40 | 0.31 | 0.16 | 0.09 | 0.09 | 0.06 | RI, Dt |
| 54 | 4-methyl-2-pentanol | 108-11-2 | C_6_H_14_O | 102.2 | 1176.7 | 405.138 | 1.54355 | 2.5 | 0.50 | 0.41 | 0.48 | 0.37 | 0.40 | 0.38 | RI, Dt |
| 55 | 3-Heptanone | 106-35-4 | C_7_H_14_O | 114.2 | 1172.4 | 399.026 | 1.58706 | 0.0075 | 0.24 | 0.20 | 0.22 | 0.17 | 0.19 | 0.18 | RI, Dt |
| 56 | 1-Penten-3-one | 1629-58-9 | C_5_H_8_O | 84.1 | 1061.9 | 274.11 | 1.08664 | 0.0009 | 1.87 | 2.51 | 2.39 | 1.93 | 2.45 | 2.48 | RI, Dt |
| 57 | Pyrrolidine | 123-75-1 | C_4_H_9_N | 71.1 | 1059.9 | 272.441 | 1.04636 | 20.2 | 1.32 | 1.05 | 1.12 | 0.97 | 1.06 | 0.86 | RI, Dt |
| 58 | 2- butanol-M | 78-92-2 | C_4_H_10_O | 74.1 | 1052.8 | 266.263 | 1.15061 | 3.3 | 0.79 | 0.75 | 0.70 | 0.78 | 1.02 | 1.08 | RI, Dt |
| 59 | 2-Pentanone | 107-87-9 | C_5_H_10_O | 86.1 | 1027.9 | 245.893 | 1.11922 | 0.01 | 1.39 | 1.28 | 1.22 | 0.68 | 0.54 | 0.61 | RI, Dt |
| 60 | Pentanal-M | 110-62-3 | C_5_H_10_O | 86.1 | 1026.6 | 244.891 | 1.18749 | 0.0027 | 1.64 | 1.55 | 1.64 | 0.97 | 1.04 | 1.03 | RI, Dt |
| 61 | 2-methyl-1-propyl acetate | 110-19-0 | C_6_H_12_O2 | 116.2 | 1024.9 | 243.542 | 1.24074 | 0.025 | 0.86 | 0.70 | 0.46 | 0.87 | 0.87 | 0.78 | RI, Dt |
| 62 | 2- butanol-D | 78-92-2 | C_4_H_10_O | 74.1 | 1041.3 | 256.696 | 1.31961 | 3.3 | 1.36 | 1.02 | 0.92 | 0.78 | 0.79 | 0.80 | RI, Dt |
| 63 | 4-Methyl-2-pentanone | 108-10-1 | C_6_H_12_O | 100.2 | 1019 | 238.988 | 1.4733 | 0.24 | 0.64 | 0.54 | 0.32 | 1.13 | 1.13 | 1.01 | RI, Dt |
| 64 | n-Pentanal-D | 110-62-3 | C_5_H_10_O | 86.1 | 1005.2 | 228.701 | 1.42881 | 0.0027 | 1.94 | 1.55 | 1.56 | 0.88 | 0.85 | 0.87 | RI, Dt |
| 65 | ethyl acrylate | 140-88-5 | C_5_H_8_O_2_ | 100.1 | 1002.4 | 226.677 | 1.39848 | 0.0001 | 1.52 | 1.28 | 1.07 | 0.89 | 0.88 | 0.94 | RI, Dt |
| 66 | 2-Propenoic acid, 2-methyl-, methyl ester | 80-62-6 | C_5_H_8_O_2_ | 100.1 | 1002.2 | 226.508 | 1.36949 | 0.05 | 1.83 | 1.56 | 0.96 | 2.52 | 1.97 | 1.89 | RI, Dt |
| 67 | 2-Ethyl furan | 3208-16-0 | C_6_H_8_O | 96.1 | 974.2 | 210.487 | 1.04931 | nd | 1.28 | 1.10 | 0.84 | 1.11 | 1.15 | 1.14 | RI, Dt |
| 68 | Ethanol | 64-17-5 | C_2_H_6_O | 46.1 | 949.5 | 198.101 | 1.14526 | 3.5 | 3.89 | 3.77 | 4.26 | 2.70 | 3.03 | 3.23 | RI, Dt |
| 69 | 2-Butanone | 78-93-3 | C_4_H_8_O | 72.1 | 951.9 | 199.23 | 1.2466 | 7 | 0.78 | 0.72 | 0.65 | 0.62 | 0.67 | 0.67 | RI, Dt |
| 70 | 1,2-Dimethoxyethane | 110-71-4 | C_4_H_10_O_2_ | 90.1 | 941.8 | 194.34 | 1.2985 | nd | 0.61 | 0.51 | 0.72 | 0.39 | 0.50 | 0.51 | RI, Dt |
| 71 | Butanal | 123-72-8 | C_4_H_8_O | 72.1 | 910.3 | 179.858 | 1.29949 | 0.002 | 1.28 | 1.14 | 1.12 | 1.00 | 1.12 | 1.04 | RI, Dt |
| 72 | 2-methyl butanal | 96-17-3 | C_5_H_10_O | 86.1 | 932.6 | 190.022 | 1.41507 | 0.0024 | 2.53 | 2.24 | 2.00 | 1.85 | 2.16 | 1.79 | RI, Dt |
| 73 | Acetic acid ethyl ester | 141-78-6 | C_4_H_8_O_2_ | 88.1 | 895.1 | 173.29 | 1.34319 | 0.005 | 3.84 | 3.45 | 3.68 | 2.62 | 3.39 | 3.02 | RI, Dt |
| 74 | 2-propanone | 67-64-1 | C_3_H_6_O | 58.1 | 832.1 | 148.387 | 1.12415 | 0.832 | 12.25 | 11.01 | 10.72 | 10.29 | 9.53 | 9.73 | RI, Dt |
| 75 | 1-octene | 111-66-0 | C_8_H_16_ | 112.2 | 855.4 | 157.142 | 1.1628 | 0.0005 | 0.54 | 0.44 | 0.30 | 0.27 | 0.23 | 0.19 | RI, Dt |
| 76 | 2,4-Dimethyl-1-heptene | 19549-87-2 | C_9_H_18_ | 126.2 | 871.2 | 163.368 | 1.20688 | nd | 0.76 | 0.67 | 0.61 | 0.53 | 0.62 | 0.43 | RI, Dt |
| 77 | Propanal | 123-38-6 | C_3_H_6_O | 58.1 | 801.7 | 137.687 | 1.14856 | 0.0092 | 2.07 | 1.73 | 1.46 | 1.57 | 1.45 | 1.58 | RI, Dt |
| 78 | Triethylamine | 121-44-8 | C_6_H_15_N | 101.2 | 765 | 125.819 | 1.09227 | 2.21 | 5.45 | 5.13 | 4.93 | 4.21 | 4.55 | 4.33 | RI, Dt |
| 79 | Cyclohexane | 110-82-7 | C_6_H_12_ | 84.2 | 734.3 | 116.675 | 1.14042 | nd | 0.79 | 0.74 | 0.75 | 0.67 | 0.87 | 0.72 | RI, Dt |
| 80 | (Z)-Ocimene | 470-82-6 | C_10_H_18_O | 154.3 | 1213.2 | 458.28 | 1.29401 | 0.000069 | 0.44 | 0.39 | 0.35 | 0.54 | 0.65 | 0.73 | RI, Dt |
| 81 | (E)-2-Pentenal-D | 1576-87-0 | C_5_H_8_O | 84.1 | 1143.3 | 360.576 | 1.36145 | 0.31 | 0.31 | 0.27 | 0.27 | 0.58 | 0.56 | 0.73 | RI, Dt |
| 82 | 2-Methyl butanoic acid ethyl ester | 7452-79-1 | C_7_H_14_O_2_ | 130.2 | 1058.7 | 271.357 | 1.23284 | 0.000006 | 0.36 | 0.31 | 0.24 | 0.47 | 0.58 | 0.59 | RI, Dt |

Contents ×10^-4^

MW; represent molecular weight

M, monomers, D, dimers

RI; Relative retention index

Rt; Retention time of VOCs in the capillary column

Dt; Drift/migration time found in the drift tube

**Table S7.** VOCs detected by GC-IMS with OAV >1

| Label | CAS# | VIP | OT(µg/g) | OAV |  |  |  |  |  |
| --- | --- | --- | --- | --- | --- | --- | --- | --- | --- |
|  |  |  |  | B2W3 | BW6 | B2W9 | B2J3 | B2J6 | B2J9 |
| 2,3-Diethyl-5-methylpyrazine | 18138-04-0 | 0.98 | 0.000031 | 1.30568 | 1.25409 | 1.25401 | 1.48139 | 1.55754 | 1.37357 |
| Pyridine | 110-86-1 | 1.17 | 0.00003 | 90.10743 | 95.20821 | 84.62214 | 67.35797 | 87.26708 | 89.50519 |
| ethyl acrylate | 140-88-5 | 0.89 | 0.0001 | 1.52237 | 1.27818 | 1.06850 | 0.89364 | 0.87704 | 0.93955 |
| 2-Methyl butanoic acid ethyl ester | 7452-79-1 | 0.98 | 0.000006 | 5.97457 | 5.12522 | 4.01185 | 7.75168 | 9.65550 | 9.81627 |
